# ES-UNet: efficient 3D medical image segmentation with enhanced skip connections in 3D UNet

**DOI:** 10.1186/s12880-025-01857-0

**Published:** 2025-08-13

**Authors:** Minyoung Park, Seungtaek Oh, Junyoung Park, Taikyeong Jeong, Sungwook Yu

**Affiliations:** 1 School of Electrical and Electronics Engineering, Chung-Ang University, 84 Heukseok-Ro, Dongjak-Gu, Seoul, 06974 Republic of Korea; 2 School of Artificial Intelligence Convergence, Hallym University, 1 Hallymdaehak-Gil, Chuncheon, 24252 Republic of Korea

**Keywords:** 3D medical image segmentation, Tumor segmentation, Data augmentation in medical imaging, UNet

## Abstract

**Background:**

Deep learning has significantly advanced medical image analysis, particularly in semantic segmentation, which is essential for clinical decisions. However, existing 3D segmentation models, like the traditional 3D UNet, face challenges in balancing computational efficiency and accuracy when processing volumetric medical data. This study aims to develop an improved architecture for 3D medical image segmentation with enhanced learning strategies to improve accuracy and address challenges related to limited training data.

**Methods:**

We propose ES-UNet, a 3D segmentation architecture that achieves superior segmentation performance while offering competitive efficiency across multiple computational metrics, including memory usage, inference time, and parameter count. The model builds upon the full-scale skip connection design of UNet3+ by integrating channel attention modules into each encoder-to-decoder path and incorporating full-scale deep supervision to enhance multi-resolution feature learning. We further introduce Region Specific Scaling (RSS), a data augmentation method that adaptively applies geometric transformations to annotated regions, and a Dynamically Weighted Dice (DWD) loss to improve the balance between precision and recall. The model was evaluated on the MICCAI HECKTOR dataset, and additional validation was conducted on selected tasks from the Medical Segmentation Decathlon (MSD).

**Results:**

On the HECKTOR dataset, ES-UNet achieved a Dice Similarity Coefficient (DSC) of 76.87%, outperforming baseline models including 3D UNet, 3D UNet 3+, nnUNet, and Swin UNETR. Ablation studies showed that RSS and DWD contributed up to 1.22% and 1.06% improvement in DSC, respectively. A sensitivity analysis demonstrated that the chosen scaling range in RSS offered a favorable trade-off between deformation and anatomical plausibility. Cross-dataset evaluation on MSD Heart and Spleen tasks also indicated strong generalization. Computational analysis revealed that ES-UNet achieves superior segmentation performance with moderate computational demands. Specifically, the enhanced skip connection design with lightweight channel attention modules integrated throughout the network architecture enables this favorable balance between high segmentation accuracy and computational efficiency.

**Conclusion:**

ES-UNet integrates architectural and algorithmic improvements to achieve robust 3D medical image segmentation. While the framework incorporates established components, its core contributions lie in the optimized skip connection strategy and supporting techniques like RSS and DWD. Future work will explore adaptive scaling strategies and broader validation across diverse imaging modalities.

## Background

In recent years, artificial intelligence (AI) has shown tremendous potential in revolutionizing various fields, including the medical industry. Specifically, medical image analysis has benefited greatly from advances in deep learning techniques, enabling automated, accurate, and efficient clinical decision-making Cireşan et al. [1], Ronneberger et al. [2], Shin et al. [3], Marullo et al. [4]. Among these advancements, medical image analysis plays a critical role in delineating anatomical structures or pathological regions, significantly aiding in lesion detection Yan et al. [5], Sherif et al. [6], cancer diagnosis Esteva et al. [7], Wu et al. [8], Zhang et al. [9], and surgical planning Twinanda et al. [10], Czempiel et al. [11], Park et al. [12, 13].

In particular, deep learning-based semantic segmentation algorithms have demonstrated remarkable success in various clinical applications, including lesion detection, cancer diagnosis, and treatment planning Loussaief et al. [14]. The most popular semantic segmentation network is UNet Ronneberger et al. [2], which is a fully convolutional neural network (CNN) that consists of a contracting path and an expanding path. The contracting path down-samples the input image to capture high-level features and produce the feature maps, whereas the expanding path up-samples the feature maps to produce the final segmentation map. By using the skip connections, UNet effectively captures fine details and small structures in an input image for accurate segmentation.

Given the success of UNet in medical image segmentation, various extensions have been proposed to further enhance its capabilities. The V-Net Milletari et al. [15] has a structure similar to UNet, as both use an encoder-decoder architecture with skip connections. However, V-Net incorporates additional residual connections within each block to improve the flow of information between layers, enhancing learning stability in deeper networks. The UNet++ Zhou et al. [16] aims to address some of the limitations of the original UNet architecture by introducing a nested U-shaped network structure with multiple skip paths at different scales. This allows the network to capture multi-scale features and improves segmentation performance compared to the original UNet. The UNet 3+ Huang et al. [17] further improves the multi-scale feature representation of UNet++ by introducing dense skip connections that connect all levels of the network. In UNet 3+, the contracting and expanding paths are connected by a dense block, enabling the network to capture fine-grained features and facilitating more efficient feature reuse. Additionally, UNet 3+ includes a feature gating mechanism that allows the network to selectively focus on important features.

Recent transformer-based architectures have also shown promising results in medical image segmentation Wu et al. [18]. Hatamizadeh et al. [19] proposed Swin UNETR, which utilizes a hierarchical Swin transformer as the encoder in a U-shaped network for brain tumor segmentation. Their approach reformulates 3D segmentation as a sequence-to-sequence prediction problem and leverages shifted windows for efficient self-attention computation, demonstrating that transformer-based architectures can effectively capture long-range dependencies critical for segmenting tumors with variable shapes and sizes. In contrast to complex architectural modifications, Isensee et al. [20] introduced nnUNet, a self-adapting framework based on vanilla U-Net architectures that automatically configures preprocessing, network topologies, training, and inference for different medical segmentation tasks. Their work emphasizes that careful optimization of non-architectural components can often yield better performance than sophisticated architectural innovations, as demonstrated by their top-ranking results across diverse datasets in the Medical Segmentation Decathlon challenge.

Despite these advancements, medical images, such as computed tomography (CT) or magnetic resonance imaging (MRI), are typically acquired as 3D volumetric data, and the methods for processing 2D data ignore the spatial correlations between adjacent slices, potentially leading to suboptimal results. Therefore, performing 3D segmentation to leverage the spatial correlations between adjacent slices is crucial for further enhancing accuracy by capturing the full volumetric context of the data. Leveraging spatial continuity between slices, 3D segmentation improves accuracy by utilizing volumetric structural information. As a result, it is important to develop efficient 3D semantic segmentation architectures, which can provide more accurate, consistent, and detailed information about anatomical structures.

Numerous 3D semantic segmentation algorithms have been developed to directly process volumetric data Hatamizadeh et al. [21], Yoo et al. [22]. For example, early models like 3D UNet Ҫiçek et al. [23] demonstrated this potential by building upon the structure of the original 2D UNet Ronneberger et al. [2] and extending its 2D operations into 3D, enabling it to handle 3D medical images directly. Despite this, their substantial computational demands limited their practical application. Studies like Zhou et al. [16], Huang et al. [17] have extended UNet with additional skip paths, but applying these to 3D models poses significant computational challenges due to the volumetric nature of 3D data and increased memory demands. In particular, UNet++ introduces additional structures between skip paths, and UNet 3+ increases the number of skip paths significantly, which can cause severe memory efficiency issues when extending these architectures from 2D to 3D. To address these challenges, efficient skip connection designs that reduce memory consumption while incorporating channel attention mechanisms Hu et al. [24] to enhance feature representation have become crucial for practical 3D medical image segmentation.

However, optimizing model architecture alone is not sufficient to overcome all challenges in medical imaging. The availability of large-scale, annotated datasets remains a major obstacle due to privacy concerns, the high cost of expert annotations, and the difficulty of acquiring medical data. This scarcity hinders the training of deep learning models, which typically require vast amounts of data to generalize well. Traditional geometric transformations such as rotation, flipping, and scaling have been widely used in medical image segmentation. However, these conventional augmentation methods often fail to introduce sufficient variability to capture the diverse anatomical variations present in clinical practice. Consequently, there is a need for data augmentation methods tailored to the unique characteristics of medical images, ensuring the integrity of anatomical structures while introducing meaningful variability.

In addition to data augmentation considerations, loss function design plays a critical role in medical image segmentation performance. Traditional loss functions such as cross-entropy loss and Dice loss employ fixed weighting schemes that may not adequately address the dynamic precision-recall trade-offs encountered during training. In medical image segmentation, class imbalance is prevalent, with target structures often occupying a small fraction of the total volume. The Dice loss, while popular for its ability to handle class imbalance, treats precision and recall equally throughout training, which may not be optimal when the model’s performance characteristics evolve during the learning process. Static weighting approaches fail to adapt to the changing needs of the model as it learns to balance between avoiding false positives and false negatives. This limitation becomes particularly pronounced in complex medical segmentation tasks where the optimal precision-recall balance may vary depending on the specific anatomical structures and clinical requirements.

To address these limitations, this paper presents ES-UNet, a novel 3D semantic segmentation architecture that adapts key concepts from 2D UNet variants to the 3D domain, with a focus on constructing efficient skip paths to enhance feature extraction. The proposed ES-UNet performs true volumetric 3D semantic segmentation, where all modules operate on full 3D image volumes rather than 2D slices, allowing the network to capture complete spatial context across all dimensions simultaneously. ES-UNet provides full-scale feature map information to all decoder blocks, supporting full-scale deep supervision and ensuring balanced predictions across all scales. Our architecture leverages enhanced full-scale skip connections with integrated channel attention modules on each encoder-to-decoder path to optimize feature representation. This design allows ES-UNet to effectively capture the most relevant features across volumetric data, achieving an optimized balance between computational requirements and segmentation accuracy.

Additionally, we introduce a new data augmentation method called Region Specific Scaling (RSS), designed to address the limitations of traditional augmentation methods in medical imaging. RSS selectively scales target regions within 3D medical images—either enlarging or reducing them along the height, width, and/or depth axes—while preserving anatomical integrity. By introducing meaningful variations in the size of anatomical features without distorting critical structures or introducing artifacts, RSS enhances data diversity and improves the model’s ability to generalize without overfitting.

Finally, we introduce a Dynamically Weighted Dice (DWD) loss function that adaptively balances precision and recall during training as detailed in Sect. DWD loss. Unlike traditional static weighting approaches, DWD dynamically adjusts the influence of precision and recall components based on the model’s current performance characteristics, ensuring a balanced approach to segmentation accuracy by minimizing both false positives and false negatives.

Through the combination of the ES-UNet architecture, the RSS augmentation method, and the DWD loss function, our approach significantly improves segmentation performance over existing methods. We achieved significantly better results compared to existing methods on the MICCAI Head and Neck Tumor Segmentation (HECKTOR) dataset Andrearczyk et al. [25], as detailed in Sect. Results. The proposed method also demonstrates a practical balance between accuracy and resource usage, with competitive inference speed and memory efficiency observed in computational comparisons. This method is versatile and can be easily adapted to other 3D image segmentation tasks.

## Methods

### ES-UNet architecture

**Fig. 1:**
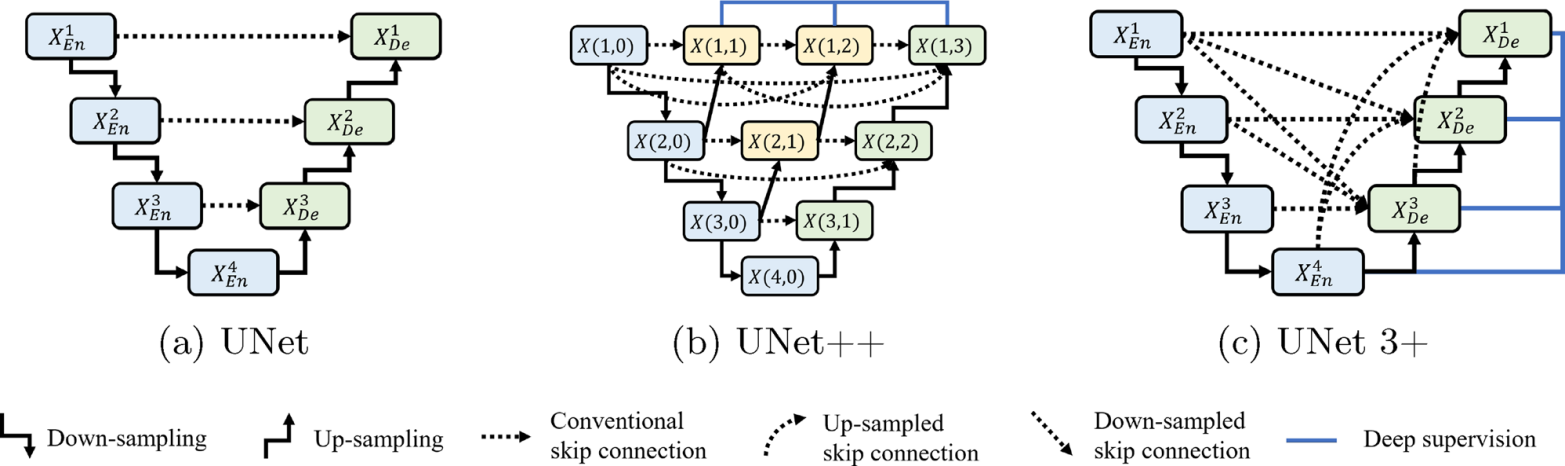
The overall structures of (**a**) UNet, (**b**) UNet++, and (**c**) UNet 3+

In the architecture of UNet, the deeper layers in the network will be referred to as “high-level” layers, where the network learns abstract, high-dimensional features. These layers lie further away from the input, possessing a larger receptive field, which allows them to capture the overall context and structure of the image. On the other hand, the “low-level” layers, located closer to the input, focus on capturing fine details and basic structural features like edges and textures. The low-level layers are crucial for capturing fine-grained details, whereas the high-level layers provide a broader understanding of the image content. Figure 1 illustrates the overall structures of the UNet variants.

In UNet, each decoder layer can only utilize the semantic information provided by skip connections at the same level, leading the network to overly rely on features from identical resolutions. UNet++ enhances the standard UNet by incorporating additional convolutional blocks within its nested skip connections, which enable multi-scale feature extraction and better capture of fine details. UNet 3+ builds upon UNet++ by adding dense skip connections that connect all levels of the network, ensuring comprehensive feature aggregation across different resolutions. However, while full-scale skip connections provide rich multi-scale information, they also present needs for optimizing skip connection paths in order to balance memory usage and feature fidelity. Without careful refinement, naive concatenation of features at every scale can cause redundant processing and unnecessary overhead, especially on high-resolution or large 3D volumes.

To effectively handle 3D medical image data, we built upon the UNet3+ architecture and incorporated an auxiliary network inspired by UNet++ (nested U-Net), implementing those additional paths as lightweight channel attention blocks. Specifically, we enhanced UNet3+’s full-scale skip connection pattern by replacing the nested convolutional modules of UNet++ with channel attention modules on each encoder-to-decoder path. This design enables efficient multi-scale feature fusion without relying on nested blocks or excessive resolution-specific skip connections. By inserting a channel attention module on every encoder-to-decoder skip path, we maintain UNet3+’s intended information flow while avoiding the large memory overhead associated with nested U-Net structures. We also applied deep supervision modules Lee et al. [26] by extending the original 2D UNet3+ scheme to our 3D decoder stages. Together, these choices allow ES-UNet to achieve accurate 3D segmentation with competitive memory usage and training efficiency.

**Fig. 2:**
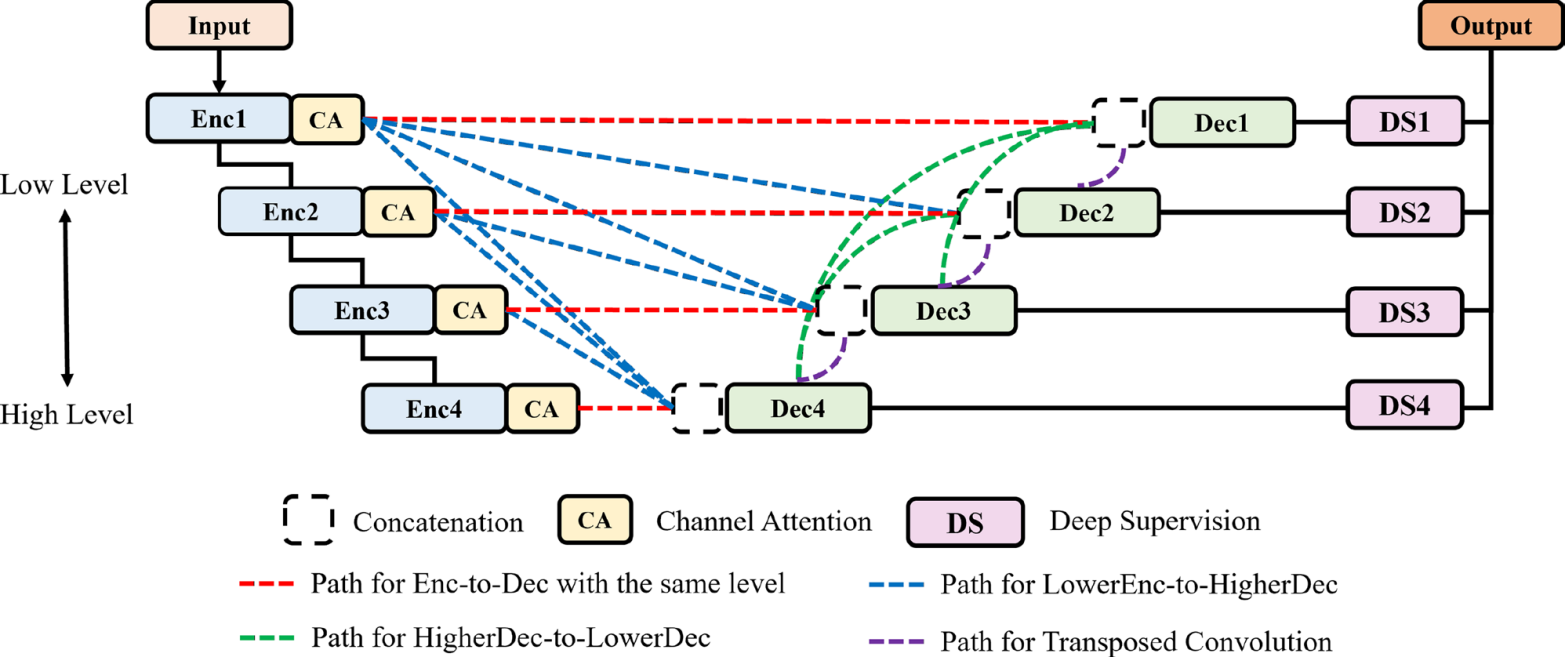
Block diagram of the proposed ES-UNet architecture

Figure 2 shows the schematic structure of ES-UNet. Details about the structure of each layer will be discussed in following sections.

#### Encoder

**Fig. 3:**
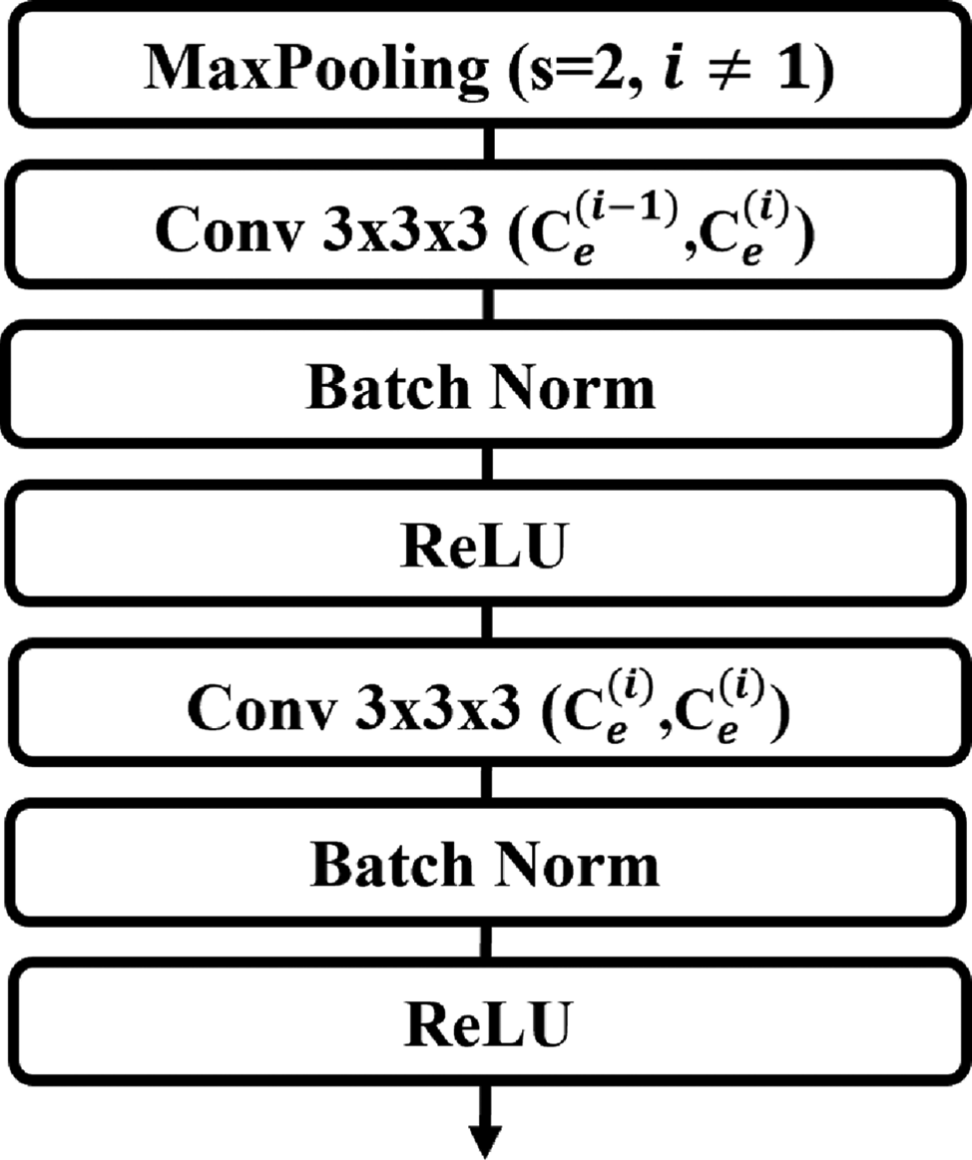
The structure of the proposed encoder block

The encoder of ES-UNet consists of $N=4$ encoder blocks. The layout of each encoder block is depicted in Fig. 3. Except for the first block, each encoder block in Fig. 2 reduces the resolution of the features from the previous block by half using a max pooling layer with a stride of 2. Each block includes two convolution layers with a kernel size of $3\times3\times3$, followed by batch normalization Ioffe and Szegedy [27] and ReLU activation Nair and Hinton [28] in sequence. The two values A and B inside Conv $3\times3\times3$ (A, B) represent the numbers of input and output channels, respectively.

The channels of each encoder block are expressed as $C_e$, where $C_e^{(i)}$ represents the channels of the $i$-th level encoder block. The input channels of the ES-UNet encoder are defined as $C_e^{(0)}$. When both PET and CT images are used, $C_e^{(0)} = 2$, whereas $C_e^{(0)} = 1$ if only one modality is used. The input image has dimensions of $\mathbb{R}^{C_e^{(0)} \times D \times H \times W}$, where $D$, $H$ and $W$ represent the depth, height and width of the input image. It should be noted that the depth, height, and width dimensions are halved during the down-sampling operation while the number of channels increases. However, this dimension reduction does not occur in the first encoder block, as it lacks a down-sampling operation. For example, if the output feature map of the first encoder block has dimensions of $\mathbb{R}^{C_e^{(1)} \times D \times H \times W}$, the output feature maps of the second, third, and fourth encoder blocks become $\mathbb{R}^{C_e^{(2)} \times \frac{D}{2} \times \frac{H}{2} \times \frac{W}{2}}$, $\mathbb{R}^{C_e^{(3)} \times \frac{D}{4} \times \frac{H}{4} \times \frac{W}{4}}$, and $\mathbb{R}^{C_e^{(4)} \times \frac{D}{8} \times \frac{H}{8} \times \frac{W}{8}}$, respectively. In our case, $C_e^{(4)}$ equals $8 \cdot C_e^{(1)}$, meaning the output feature map of the ES-UNet encoder will have dimensions of $\mathbb{R}^{8 \cdot C_e^{(1)} \times \frac{D}{8} \times \frac{H}{8} \times \frac{W}{8}}$.

We built ES-UNet on the UNet3+ backbone, which already employs full-scale skip connections. Drawing inspiration from UNet++’s nested concatenation design, we aimed to enhance each skip path by adding lightweight refinement modules. However, directly inserting extra convolutions or complex blocks into every skip connection would have imposed a significant computational burden. To address this, we adopted a channel attention module from Squeeze and Excitation Networks (SENets) Hu et al. [24] and applied it only on the encoder-to-decoder skip paths rather than between encoder blocks. In this setup, the reduction ratio for all channel attention layers was uniformly set to 0.25, following the original SENet design. By doing so, we improve feature integration along the skip connections while keeping the overall complexity of the network manageable.

#### Decoder

**Fig. 4:**
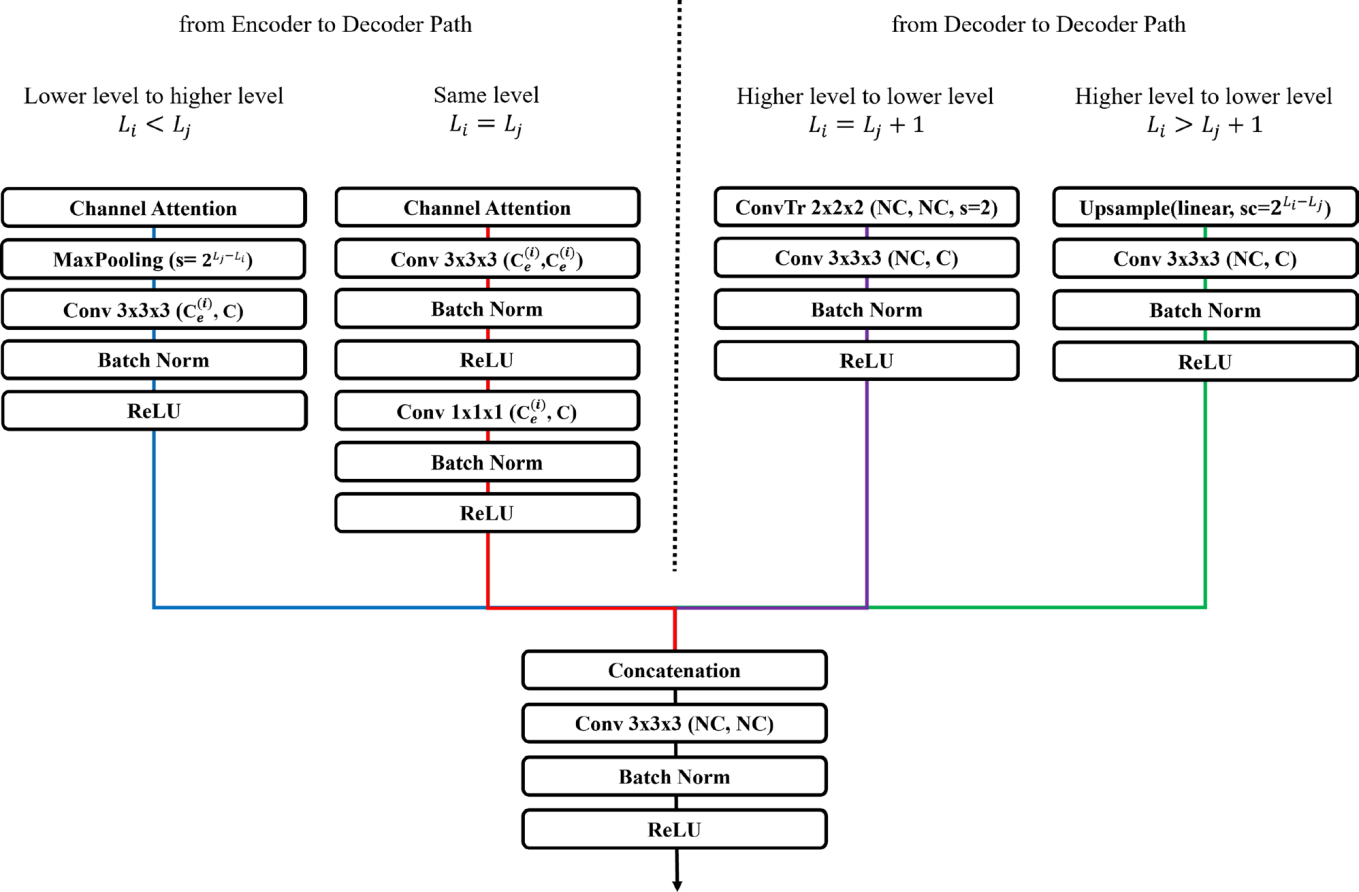
The structure of the proposed decoder block

The decoder of ES-UNet is also composed of a total of $N=4$ decoder blocks similar to the encoder. In the decoder blocks, different methods are applied to process data based on the level from which the input feature maps originate. Figure 4 shows the proposed structure of the decoder block. The colors of the paths in Fig. 4 follow and correspond to the colors of the respective paths in Fig. 3, ensuring consistency in visual representation.

In the figure, $L_{i}$ represents the level of the encoder/decoder that produces the current decoder’s input feature map, while $L_{j}$ represents the level of the current decoder. In a generic encoder–decoder architecture, there are three possible ways to connect an encoder feature map at level $L_i$ to a decoder block at level $L_j$: (1) $L_i < L_j$, i.e., routing a higher-resolution feature down to a lower-resolution decoder; (2) $L_i = L_j$, i.e., the same-level skip connection; (3) $L_i > L_j$, i.e., routing a lower-resolution encoder output up to a higher-resolution decoder. UNet3+ implements “full-scale” skip connections by covering cases (1) and (2) for all $i, j$ such that $i \le j$, while it does not include direct connections for $i > j$. In ES-UNet, we follow the same full-scale design (i.e., we keep all $i \le j$ connections), but we replace UNet++’s nested convolutional blocks with lightweight channel attention modules on each encoder-to-decoder path.

First, we examine the scenario in which the output from the encoder block is directed to a higher level in the decoder, which occurs when $L_{i} < L_{j}$. The output feature map from the encoder block is first passed through a channel attention layer and then down-sampled to the appropriate resolution for the decoder level with a max pooling layer. It then undergoes a $3\times3\times3$ convolution layer to adjust the number of channels to $C$, which is a value set for each level in the decoder, ensuring that all levels are weighted equally during the processing. As with the encoder, all convolution layers in the decoder (excluding transposed convolution layers) are followed by Batch Normalization and ReLU activation in sequence.

The other scenario in the encoder-to-decoder path occurs when the encoder level and decoder level are the same, $L_{i} = L_{j}$. In this case, the output from the encoder block, after passing through a channel attention layer, is processed through two $3\times3\times3$ convolution layers and one $1\times1\times1$ convolution layer to produce feature maps with $C$ channels.

Subsequently, we examine the scenarios in which data flows between different decoder blocks, where two distinct cases arise. The first scenario involves the typical up-sampling path found in decoders, where only one level is reduced, as when $L_{i}$ equals $L_{j} + 1$. In this case, up-sampling is performed using a $2\times2\times2$ transposed convolution with a stride of 2, followed by a $3\times3\times3$ convolution layer to adjust the channels to $C$. In the second scenario, where $L_{i}$ is greater than $L_{j} + 1$, up-sampling is carried out using a trilinear interpolation layer instead of a transposed convolution, to match the resolution required for the corresponding decoder level. The output channels are adjusted to $C$ through a $3\times3\times3$ convolution layer.

Each decoder block then gathers the feature maps from the corresponding level’s paths and concatenates them. After concatenation, the feature maps are passed through a $3\times3\times3$ convolution layer to process multi-level information. Since each decoder block is connected to $N$ paths, the input channels for the convolution layer after concatenation become $NC$. To maintain consistency in the model size across all deep supervision networks, the output channels are also standardized to $NC$.

Unlike our baseline 3D UNet3+ implementation, which relies solely on parameter-free trilinear interpolation for upsampling inside the decoder, ES-UNet uses learnable transposed convolution layers for upsampling between neighboring decoder stages. By using transposed convolution layers, the network learns upsampling filters that better recover fine structural details and object boundaries—improvements that static trilinear interpolation cannot achieve. Although learnable transposed convolution layers introduce additional parameters and increase computational cost, the gain in boundary accuracy and small-structure segmentation generally outweighs this overhead, particularly since we have reduced complexity in other parts of the network.

#### Auxiliary network for deep supervision

**Fig. 5:**
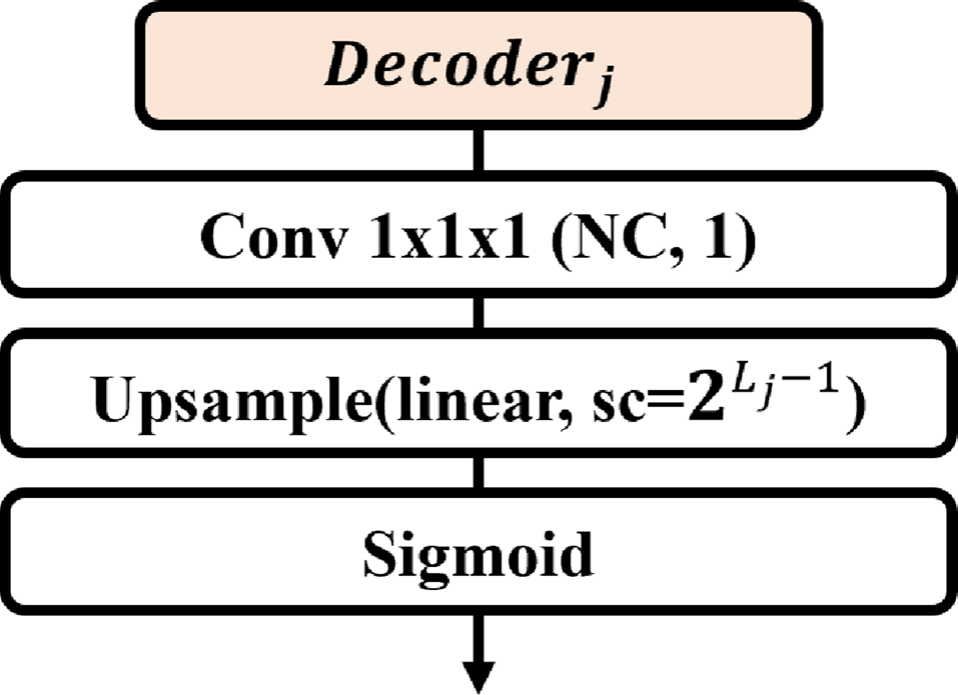
The structure of the $j$-th auxiliary network for deep supervision

To facilitate deep supervision within the ES-UNet architecture, we integrate auxiliary networks directly after each decoder layer, as illustrated in Fig. 5. These auxiliary networks are designed to enhance the learning process by providing additional gradient signals during training, thus reinforcing the feature extraction capabilities at each decoding stage.

Each auxiliary network is composed of a series of operations: a $1\times1\times1$ convolution layer that condenses the feature maps, followed by a trilinear interpolation up-sampling layer, which scales the features up to the resolution of the original input image. The scaling factor for the up-sampling layer is set to $2^{L_{j} - 1}$ ensuring that the output resolution precisely matches that of the initial input, thereby preserving spatial coherence across all levels of the network. The final operation within these auxiliary networks is a sigmoid activation function, which refines the feature maps into probability maps, suitable for the final segmentation tasks.

Upon completion of the deep supervision process, the outputs from each auxiliary network are combined to generate the final prediction. This is accomplished by calculating the equally weighted arithmetic mean of the outputs from all deep supervision networks. This averaging method not only ensures that the contributions from all levels of the decoder are harmoniously integrated but also enhances the robustness and accuracy of the segmentation results. By leveraging deep supervision in this manner, the ES-UNet architecture is able to achieve superior performance, particularly in scenarios that demand precise, multi-scale feature integration.

### Region specific scaling

As is well known, it is not easy to obtain training data in the field of medical imaging, especially in the field of 3D image applications. To solve this problem, we propose a new data augmentation method, Region Specific Scaling (RSS). The proposed RSS method either increases or reduces the target region in the direction of height, width, and/or depth.

In medical imaging, particularly in CT scans, the human body is typically visualized in three main planes: axial, sagittal, and coronal. An axial image represents a horizontal slice of the body, taken parallel to the ground, and is commonly viewed from the top down, as if looking at the body from above. This plane provides a cross-sectional view, showing structures from head to toe. A sagittal image represents a vertical slice from side to side, giving a profile view of the body. A coronal image shows a vertical slice from front to back, offering a view as if looking directly at the person from the front.

**Fig. 6:**
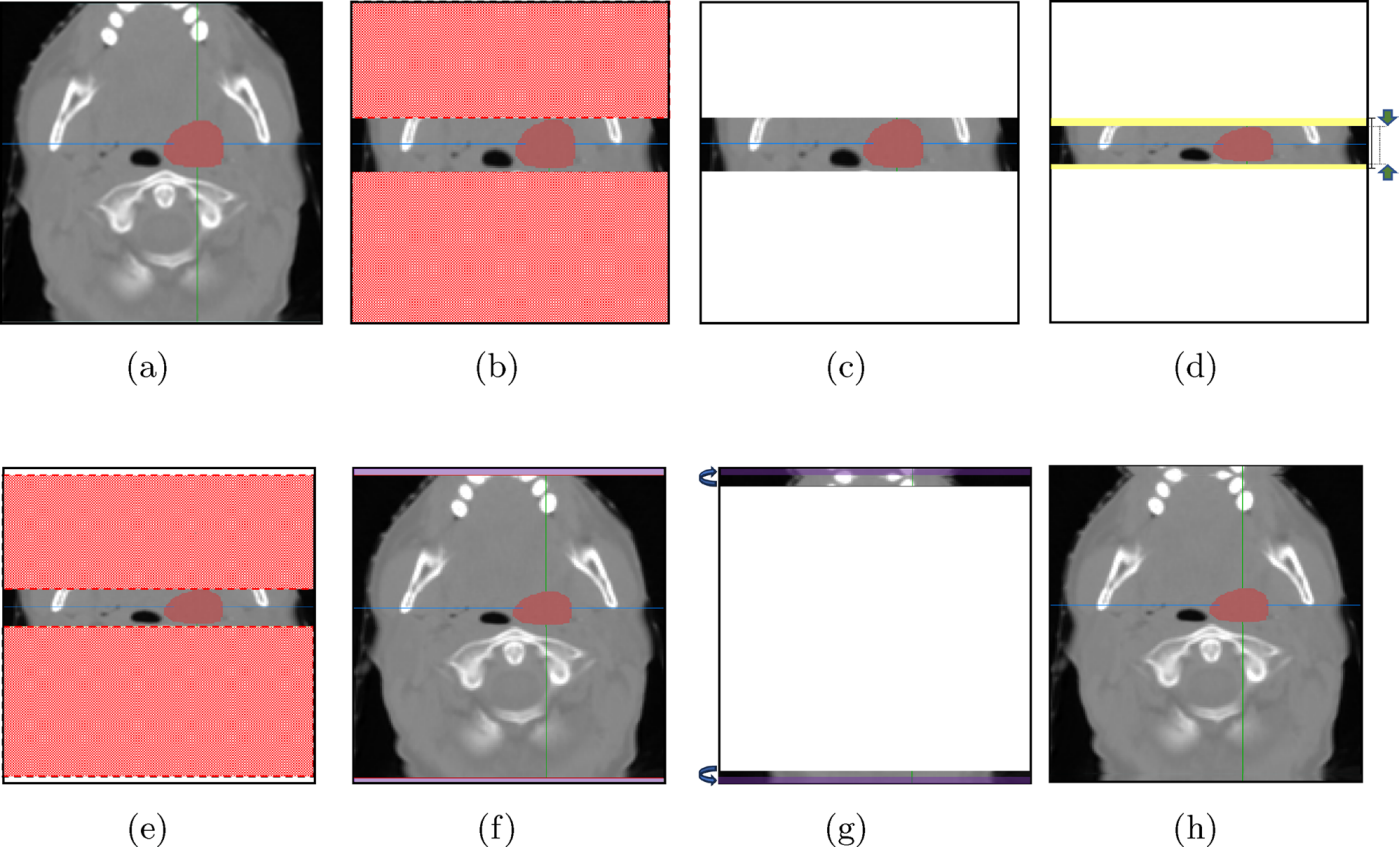
An illustration of region reduction along the height axis in an axial image by the RSS method

To aid in understanding how our approach operates, we will explain the method in detail using two specific cases. The first case involves down-scaling along the height direction in an axial image, while the second case involves up-scaling along the same direction. Figure 6 shows an example of the first case where the target region of a sample image is reduced in the direction of height.

Figure 6a shows an axial image (i.e., a slice taken along the depth axis) from a CT scan. The proposed RSS method scales this input image in the direction of height as follows. First, the RSS method identifies the minimum and maximum heights of the target label area, which is shown in red color in Fig. 6a. That is, the RSS method identifies the range of the labeled region by finding the maximum and minimum y-indices of all voxels with a non-zero label value (i.e., label = 1). These minimum and maximum values define the boundaries of the target region for scaling operations along that particular axis. Importantly, this process is performed across all slices of the 3D volume to ensure that the entire labeled structure is captured within the target region. The global minimum and maximum coordinates from all slices are used to define the comprehensive target area boundaries, rather than processing each slice individually. By using these values, the RSS method divides the original input image into 3 parts, as shown in Fig. 6b. Then, both the upper and lower rectangle areas in Fig. 6b are removed temporarily, as shown in Fig. 6c. The remaining region (i.e., the middle region in Fig. 6b) is scaled using an arbitrary ratio $r$ in the range of [$r_1$, 1], as shown in Fig. 6d. The value of $r_1$ is set to $2/3$ so that it can introduce sufficient variability without distorting anatomical structures in a way that would impede the model’s learning. Then, the two rectangles that were temporarily removed in Fig. 6c are reattached, as shown in Figs. 6e and 6f. Thus, the two rectangle areas are not subject to any scale transform. It should be noted that there will be blank areas in both the top part and the bottom part of Figs. 6e and 6f because of the reduction process performed in Fig. 6d. The proposed RSS method uses the mirror padding technique, as shown in Fig. 6g to effectively fill these gaps, ensuring the continuity and integrity of the image. Figure 6h shows the final image that will be used in the training process of the proposed method. Although Fig. 6 shows an example where the input image is scaled along the height direction, the proposed RSS method can be applied along any of the three axes: height, width, or depth. That is, this target region identification process is performed separately for each axis since the proposed RSS technique applies scaling independently along each of the three anatomical axes (axial, sagittal, and coronal). This allows for axis-specific scaling that respects the unique spatial characteristics of the anatomical structure in each dimension.

**Fig. 7:**
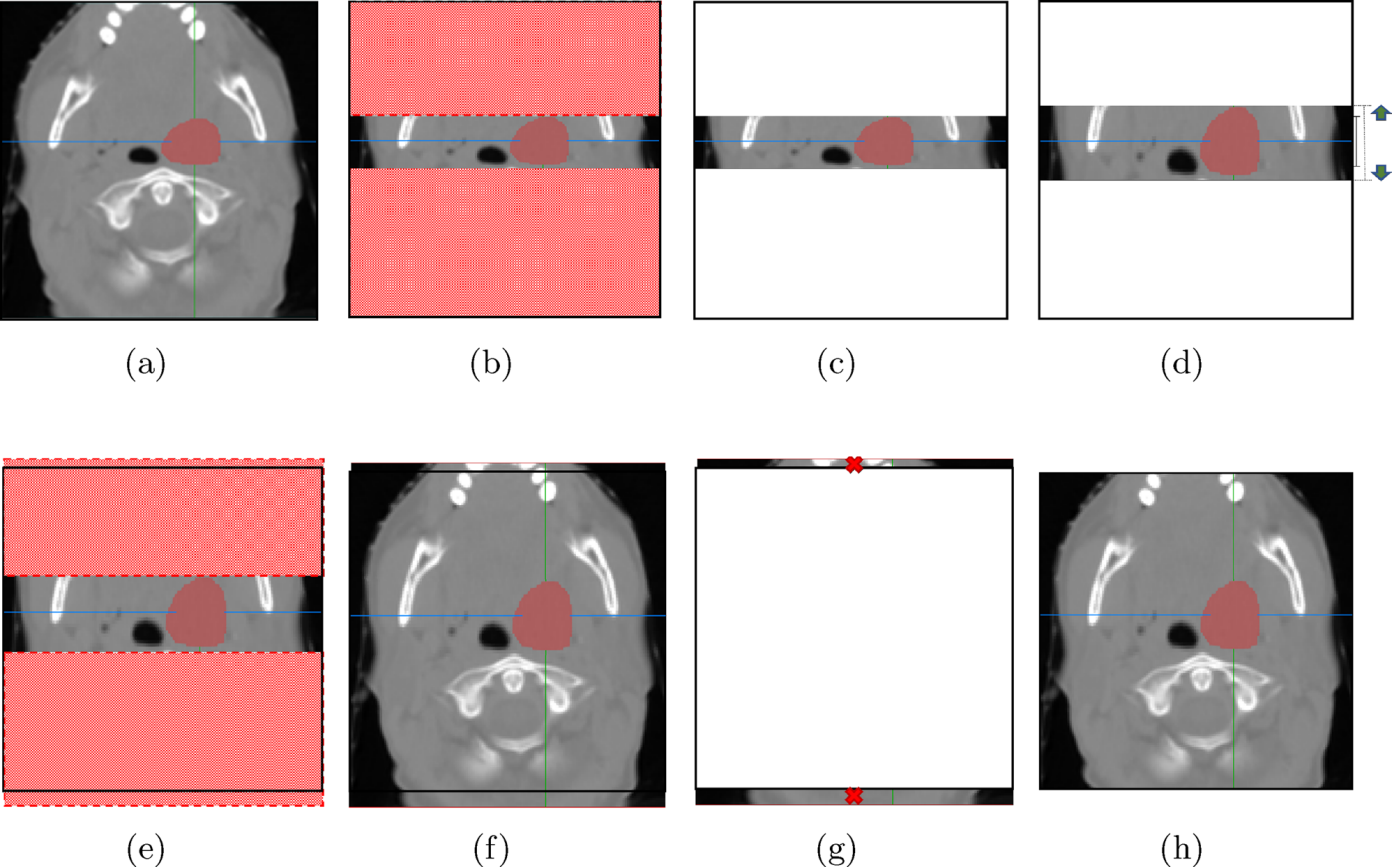
An illustration of region enlargement along the height axis by the RSS method

Figure 7 shows an example of the second case, where the target region of a sample image is enlarged (instead of being reduced) in the direction of height. This example follows the same process as Fig. 6, with the key difference being the scale enlargement, which results in a slightly altered procedure. The scaling (i.e., enlargement in this case) operation is performed using an arbitrary ratio $r$, which is randomly sampled from the range [1, $r_2$], where the value of $r_2$ is set to $3/2$, as shown in Fig. 7d. Once again, the value of $r_2$ is chosen to introduce sufficient variability without distorting anatomical structures. As in the previous example, the regions highlighted in red are not subject to any scale transform. As a result, the size of the intermediate image after reattachment will exceed the original size, as shown in Figs. 7e and 7f. This excess area will simply be removed, as shown in Fig. 7g, resulting in the final training image, shown in Fig. 7h.

In general, RSS can be applied independently to the height, width, and/or depth axes, but this operation is performed sequentially rather than simultaneously. The probability of applying RSS to each axis is set equally, ensuring independent application across all axes. The overall probability of applying RSS at least once on any axis is set to $p_{RSS} = 1 - (1 - p_x)(1 - p_y)(1 - p_z) = 0.5$. Therefore, $p_x = p_y = p_z = 1 - \sqrt[3]{0.5} \approx 0.2063$.

### DWD loss

To improve its capabilities even further, we propose new types of loss functions. Our approach involves blending the strengths of pixel-based loss functions and region-based loss functions to create a well-rounded and advanced loss function. One of the most popular pixel-based loss function is the focal loss Lin et al. [29], which is shown below.

1 $$\begin{aligned}\mathcal{L}_{\mathrm{focal}}(y_i, p_i) =& -\bigl(1 - y_i p_i - (1 - y_i)(1 - p_i)\bigr)^{\gamma}\,\\&\log\bigl(y_i p_i + (1 - y_i)(1 - p_i)\bigr)\end{aligned}$$

In Eq. 1, $y_i \in \{0, 1\}$ represents the ground truth value for the $i$-th voxel, where $i \in \{1, 2, \dots, V\}$. Here, $V$ represents the total number of voxels. The prediction probability for the $i$-th voxel is denoted by $p_i$, and $\gamma \in [0, 5]$ is a tunable focusing parameter. We adopted $\gamma = 2$, following the recommendation in Lin et al. [29], where this value was shown to effectively address class imbalance by focusing more on hard-to-classify examples. Focal loss is an enhanced version of cross-entropy loss (CEL) and it offers a smart way to address a common issue in segmentation tasks – the imbalance between easy and hard examples. By giving more attention to challenging examples, focal loss helps the model better focus on regions that are typically harder to classify.

Meanwhile, one of the most popular region-based loss functions is the Dice loss function Milletari et al. [15], which is shown below.

2 $$\mathcal{L}_{\mathrm{Dice}} = 1 - \frac{2\,\sum_{i=1}^{V}y_i p_i} {\sum_{i=1}^{V}y_i^2 + \sum_{i=1}^{V}p_i^2}$$

Dice loss measures the similarity between predicted and ground truth masks and it focuses on the intersection between these masks while also considering their individual areas. Dice loss is particularly effective in scenarios where class imbalances exist, as it helps address the challenges posed by unevenly distributed pixels. By encouraging higher values for accurate predictions and penalizing false negatives, Dice loss promotes more precise segmentation outcomes in medical imaging and other related tasks. In this paper, we propose a new region based loss function, called Dynamically Weighted Dice (DWD) loss, which is defined as:

3 $$\mathcal{L}_{\mathrm{DWD}} = 1 - \frac{2\,\sum_{i=1}^{V}y_i p_i} {\,w_P\,\sum_{i=1}^{V}y_i^2 + w_R\,\sum_{i=1}^{V}p_i^2}$$

where $w_P$ and $w_R$ are weighting factors given by:

4 $${w_P} = 1{\mkern 1mu} - {\mkern 1mu} ({C_P} - {C_R}),\,\,{w_R} = 1{\mkern 1mu} - {\mkern 1mu} ({C_R} - {C_P}),$$

where $C_P$ and $C_R$ represent the precision and recall components, respectively, and are defined as:

5 $${C_P} = \frac{{\sum\limits_{i = 1}^V {{y_i}} {p_i}}}{{\sum\limits_{i = 1}^V {p_i^2} }},\,{C_P} = \frac{{\sum\limits_{i = 1}^V {{y_i}} {p_i}}}{{\sum\limits_{i = 1}^V {y_i^2} }}.$$

In Equation 5, the precision coefficient $C_P$ represents the ratio of correctly predicted voxels among all the voxels predicted as objects, while the recall coefficient $C_R$, denotes the ratio of voxels predicted as objects among all the ground truth voxels. It should be noted that $C_P$ is different from the regular “precision” in that $p_i$ in $C_P$ can take any value between [0, 1], whereas $\hat{y}_i$ in “precision” takes the value of either 0 or 1. For the same reason, $C_R$ is different from the regular “recall”.

It should be noted that the proposed DWD loss degenerates to the conventional Dice loss when $C_P = C_R$ (since $w_P = w_R = 1$). However, DWD loss improves upon Dice loss by dynamically adjusting the influence of $C_P$ and $C_R$ when there is a disparity between them. This adjustment enables DWD loss to automatically balance precision and recall, yielding more robust performance when $C_P$ and $C_R$ diverge. Specifically, when $C_P < C_R$ (i.e., precision is lower than recall), the weights become $w_P = 1 + (C_R - C_P) > 1$ and $w_R = 1 - (C_R - C_P) < 1$, thereby penalising false positives more strongly. Conversely, when $C_R < C_P$, the roles are reversed. This mechanism amplifies the contribution of the coefficient with the larger value, thereby balancing the influence of both coefficients. As a result, when there is a significant disparity between $C_P$ and $C_R$, the DWD loss $\mathcal{L}_{DWD}$ is magnified, reflecting the increased importance of balancing precision and recall in such cases. The final loss function of the proposed method is given as follows, where $\alpha$ is a coefficient that controls the relative significance between the focal loss component and the DWD loss component. A higher $\alpha$ value increases the influence of the DWD loss (which focuses on region-based segmentation accuracy), while a lower $\alpha$ value emphasizes the focal loss (which addresses pixel-wise classification). In our implementation, we set $\alpha = 1$ based on preliminary experiments, and the effect of different $\alpha$ values on segmentation performance will be analyzed in the ablation study section.

6 $$\mathcal{L}_{\mathrm{total}} = \frac{1}{V}\,\sum_{i=1}^{V}\mathcal{L}_{\mathrm{focal}}(y_i, p_i) \,+\,\alpha\,\mathcal{L}_{\mathrm{DWD}}$$

It should be noted that each component contributes a unique aspect to the overall strategy. In particular, the DWD loss enriches the proposed ES-UNet framework by intelligently weighting coefficients based on their relative values. This augmentation fosters improved segmentation outcomes, particularly in scenarios characterized by substantial differences between precision and recall. The effect of this new loss function will be discussed in more detail in Sect. Effect of the DWD loss function.

The dynamic nature of DWD provides several key advantages over static weighting schemes. First, it eliminates the need for manual tuning of weighting parameters, which can be dataset-dependent and time-consuming. Second, it allows the loss function to adapt to different phases of training - for example, when a model initially performs well on precision but struggles with recall, the DWD loss automatically increases the influence of recall-related terms, steering optimization toward a more balanced outcome. Third, by operating at the regional level rather than the pixel level, DWD addresses class imbalance in a more comprehensive manner than pixel-wise weighting approaches.

These characteristics make DWD particularly effective for medical image segmentation tasks where capturing precise boundaries of anatomical structures is crucial, and where the trade-off between precision and recall can significantly impact clinical utility. The experimental results presented in Sect. Effect of the DWD loss function demonstrate that this dynamic adaptation mechanism consistently improves segmentation performance across different architectures.

## Results

### Training configurations

**Fig. 8:**
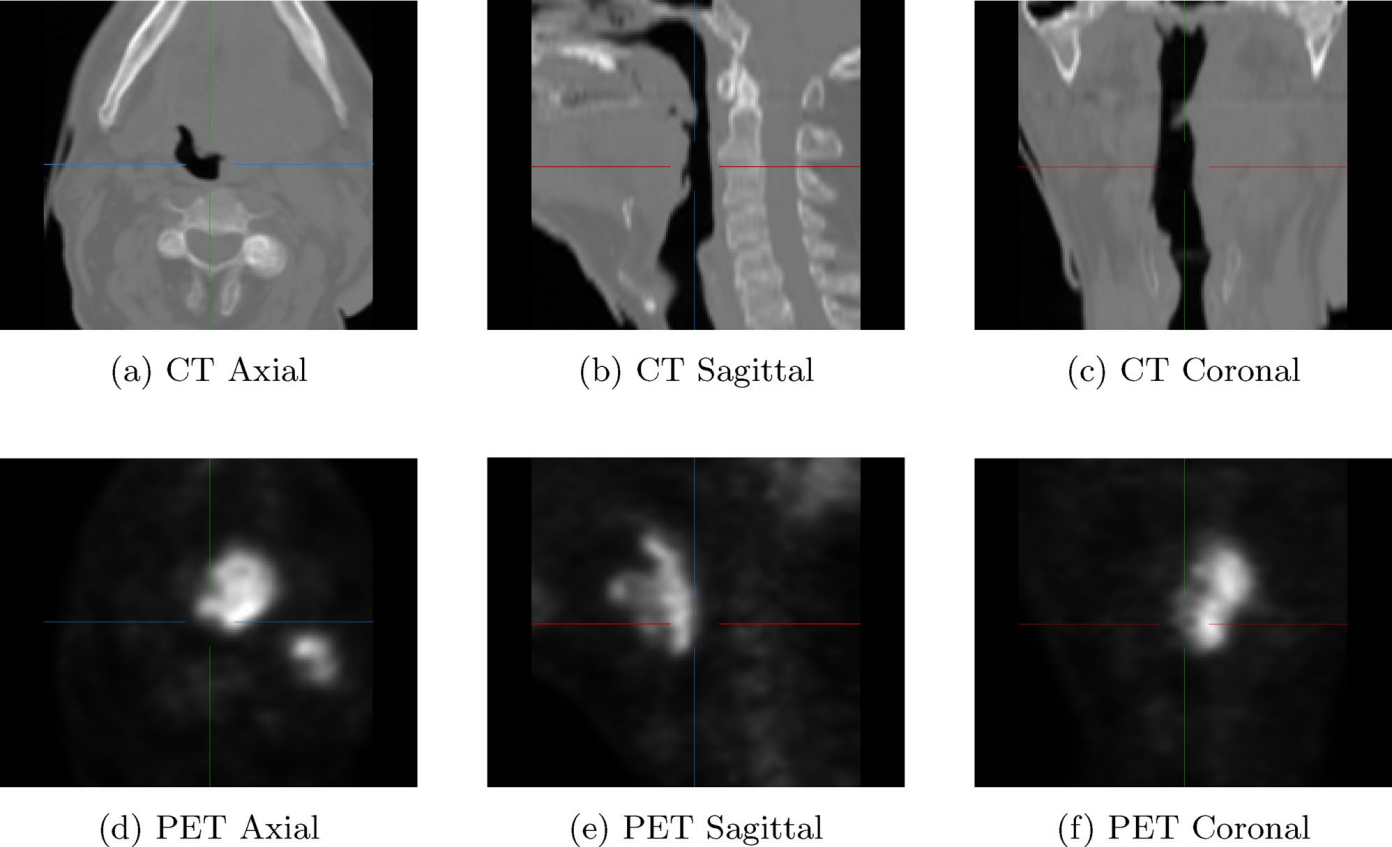
Axial, sagittal, and coronal views of the CT (top row) and PET (bottom row) images from the HECKTOR dataset. These images provide a comprehensive visualization of the anatomical and metabolic information, with the CT scans highlighting the structural details and the PET scans showing metabolic activity within the same regions

The ES-UNet has been validated on the MICCAI HECKTOR dataset Andrearczyk et al. [25]. The Head and Neck dataset consists of 325 pairs (i.e., CT and PET image pairs) of 3D images, of which 224 pairs were used as the training dataset and 101 pairs as the test dataset, respectively. The 224 labelled training pairs were randomly divided into 180 cases for optimization and 44 cases ($\approx$20%) reserved for evaluation. Both CT and PET images have a size of $144\times144\times144$ voxels. Figure 8 shows a pair of CT and PET image data from the HECKTOR dataset. During both training and inference, the model processes full 3D volumes without slicing them into 2D sections. Evaluation metrics are computed based on 3D ground truth labels and 3D predictions, ensuring a true volumetric assessment of performance.

To compensate for the limited size of the HECKTOR dataset and enhance the model’s ability to generalize, several data augmentation techniques were applied during training. First, mirroring was applied with a probability of 0.5, and rotation by a random angle between −15$^{\circ}$ and 15$^{\circ}$ was performed in each axial direction. Additionally, the RSS was employed to transform the size of the target area by a factor $r$ in the range of [$r_1$, $r_2$] = [$2/3$, $3/2$] for each of the depth, height, and width axes. As mentioned in Sect. Region specific scaling, the values of $r_1$ and $r_2$ were chosen to maintain consistency and ensure balanced scaling across both dimensions. By using these ratios, we effectively mimic real-world variations in anatomical sizes while maintaining a realistic representation of the medical images. The Adam optimizer Kingma [30] was employed to optimize the model’s performance. The hyperparameters $(\beta_1, \beta_2)$, which control the decay rates of the moving averages for the gradients (first moments) and the squared gradients (second moments), were set to 0.9 and 0.99 respectively, following the recommendations in Kingma [30].

To ensure a more effective and adaptive learning process, a learning rate scheduler was applied to dynamically adjust the learning rate throughout training. Specifically, the cosine annealing warm restarts method Loshchilov and Hutter [31] was used, where the learning rate started at 1e-3 and was gradually reduced with warm-up restarts every 25 epochs to a minimum of 1e-5, over a total of 100 epochs of training.

To evaluate the segmentation performance, we used three standard metrics: the Dice Similarity Coefficient (DSC), the Intersection over Union (IoU), and the Volume Overlap Error (VOE). The DSC is calculated as the ratio of twice the area of overlap between the predicted segmentation map $\hat{Y}$ and the ground truth $Y$ to the sum of their areas:

7 $$ \text{DSC} = \frac{2 \cdot|\hat{Y} \cap Y|}{|\hat{Y}| +|Y|}$$

The IoU, also known as the Jaccard index, is defined as the ratio of the intersection to the union of the predicted and ground truth segmentations:

8 $$ \text{IoU} = \frac{|\hat{Y} \cap Y|}{|\hat{Y} \cup Y|}$$

The Volume Overlap Error (VOE) is derived from IoU and represents the proportion of non-overlapping volume between the prediction and ground truth:

9 $$ \text{VOE} = 1 - \text{IoU}$$

These metrics together provide a comprehensive assessment of segmentation accuracy, with DSC emphasizing similarity, IoU capturing proportional overlap, and VOE offering an error-based interpretation that highlights the degree of volumetric mismatch.

### Simulation results for the ES-UNet architecture

The ES-UNet architecture described in Sect. ES-UNet architecture has a structure similar to the 3D UNet and the UNet 3+ implemented in 3D, which we refer to as 3D UNet 3+, but incorporates several architectural enhancements to improve performance. Table 1 compares the basic 3D UNet, the 3D UNet 3+, and the proposed ES-UNet in terms of DSC, IoU and VOE (Volume Overlap Error) metrics. To ensure a fair comparison that focuses solely on the architectural differences, all other experimental conditions were kept consistent across models. For instance, all three architectures were trained using identical data augmentation techniques, with the exception of the RSS method in this case. For the loss function, a combination of the focal loss in Eq. 1 and the Dice loss in Eq. 2 was used for all three architectures.

**Table 1:** Comparison of 3D UNet, 3D UNet 3+, and the proposed ES-UNet in terms of DSC, IoU and VOE

Model	DSC (%$\uparrow$)	IoU (%$\uparrow$)	VOE (%$\downarrow$)
3D UNet Ҫiçek et al. [23]	73.81	62.27	37.73
3D UNet 3+ Huang et al. [17]	74.74	63.23	36.77
ES-UNet (ours)	**75.38**	**63.61**	**36.39**

The performance gains of ES-UNet stem from its enhanced skip connection strategy, which incorporates every encoder-to-decoder path from UNet3+ with a dedicated channel attention layer on each path. Additionally, learnable transposed convolution layers between adjacent decoder stages improve upsampling quality compared to standard interpolation methods. These components work together to highlight the most relevant features at each resolution and recover fine boundary details, ensuring that all decoder stages benefit from attended multi-scale information and effective deep supervision across every level.

### Effect of the region specific scaling technique

Effective data augmentation techniques can increase the diversity of training data, thereby reducing the risk of overfitting. Table 2 presents the DSC values of 3D UNet, the 3D UNet 3+, and ES-UNet, both with and without the proposed RSS, in addition to mirroring and rotation methods. The results show that RSS improves performance across all three models. This improvement is likely due to the proposed method’s ability to enhance data diversity and reduce the risk of overfitting.

**Table 2:** Comparison of DSC, IoU and VOE with and without RSS

Model	Mirror & Rotate			Mirror & Rotate + RSS			Difference
	DSC (%$\uparrow$)	IoU (%$\uparrow$)	VOE (%$\downarrow$)	DSC (%$\uparrow$)	IoU (%$\uparrow$)	VOE (%$\downarrow$)	$\Delta$ DSC (%$\uparrow$)
3D UNet Ҫiçek et al. [23]	73.81	62.40	37.60	75.03	63.57	36.43	**1.22**
3D UNet 3+ Huang et al. [17]	74.74	63.23	36.77	75.17	63.94	36.06	0.43
ES-UNet	**75.38**	**63.61**	**36.39**	**75.74**	**64.59**	**35.41**	0.36

It should be noted that the RSS data augmentation method played an important role in enhancing model performance by ensuring that the augmented samples were more distinct from the original data. Traditional augmentation methods, such as flipping and rotation, do not significantly alter image patterns, so the augmented samples often appear largely unchanged to human observers. This limited variability can restrict the model’s ability to learn effectively from these samples. In contrast, RSS introduces targeted transformations that adjust the inherent proportions of the sample, which makes these modified samples appear as entirely new variations. For instance, adjusting the distance between anatomical features in a medical image can significantly alter the model’s interpretation, much like how changing facial proportions in a photo could make someone look like a different person. By applying the controlled range of scaling ratios $2/3$ to $3/2$, RSS increases data diversity without creating unrealistic samples. This sensible range ensures that the transformations are plausible and realistic, thereby preventing distortion of real-world anatomical structures. This balanced approach likely contributed to the improved model performance by exposing the model to a wider variety of meaningful, realistic variations.

### Effect of the DWD loss function

In Sect. DWD loss, we proposed a new loss function, called DWD loss, as a potential replacement for the conventional Dice loss. Table 3 compares the performance of these two loss functions using the DSC metric. It is important to note that in both cases, a focal loss component was included as a pixel-based loss function. The DSC value for the 3D UNet model increased from 73.81% to 74.87% with the introduction of DWD loss. Similarly, in 3D UNet 3+ and the proposed ES-UNet, the DSC values improved by 0.77% and 0.64%, respectively, confirming that DWD loss contributes to the overall performance improvement of the models.

**Table 3:** Comparison of DSC, IoU and VOE using dice loss and DWD loss

Model	Dice loss			DWD loss			Difference
	DSC (%$\uparrow$)	IoU (%$\uparrow$)	VOE (%$\downarrow$)	DSC (%$\uparrow$)	IoU (%$\uparrow$)	VOE (%$\downarrow$)	$\Delta$DSC (%$\uparrow$)
3D UNet Ҫiçek et al. [23]	73.81	62.40	37.60	74.87	63.42	36.58	**1.06**
3D UNet 3+ Huang et al. [17]	74.74	63.23	36.77	75.47	64.02	35.98	0.73
ES-UNet	**75.38**	**63.61**	**36.39**	**76.02**	**64.73**	**35.27**	0.64

The Dice loss, being symmetric, treats false positives and false negatives equally, which might not always be ideal depending on the application. This approach can sometimes limit the model’s ability to handle specific challenges, such as class imbalance or complex segmentation boundaries, where focusing on either precision or recall might be more beneficial. The DWD loss, however, adapts dynamically to the relative importance of precision and recall. This adaptability allows the loss function to shift its focus to areas where the model requires more improvement. For example, if the model’s recall is lower than its precision, the weighting factors ($w_P$ and $w_R$) will emphasize recall more, helping the model learn more effectively from its mistakes. This flexibility helps the model avoid overfitting to specific types of errors (e.g., focusing solely on minimizing false negatives while ignoring false positives) and promotes a more generalized learning approach.

### Ablation study

#### Skip connection ablation study

To provide a comprehensive understanding of the ES-UNet architecture design, we conducted a detailed ablation study examining the individual contributions of different skip connection types. As illustrated in Fig 2, our ES-UNet employs two distinct types of skip connections: encoder-to-decoder (Enc-Dec) connections (shown in blue dashed lines) and decoder-to-decoder (Dec-Dec) connections (shown in green dashed lines), in addition to the conventional same-level skip connections (shown in red solid lines).

Table 4 presents the systematic evaluation of the following four different skip connection configurations:

- Base - using only conventional same-level skip connections
- Enc-only - adding encoder-to-decoder skip connections between different levels
- Dec-only - adding decoder-to-decoder skip connections
- Both - the complete ES-UNet architecture with all skip connection types

**Table 4:** Ablation study of skip connections in ES-UNet

Configuration	Enc–Dec	Dec–Dec	DSC	IoU	VOE
			(%$\uparrow$)	(%$\uparrow$)	(%$\downarrow$)
Base			73.88	62.26	37.74
Enc-only	$\checkmark$		74.59	63.02	36.98
Dec-only		$\checkmark$	74.68	**63.72**	**36.28**
Both	$\checkmark$	$\checkmark$	**75.38**	63.61	36.39

The results demonstrate a clear performance hierarchy, with the complete ES-UNet configuration (shown as “Both”) achieving the highest DSC of 75.38%, followed by configurations using individual skip connection types, and the “Base” configuration (using only same-level skips) showing the lowest performance. The encoder-to-decoder skip connections (Enc-only configuration) enable each decoder layer to receive feature maps from multiple encoder levels. This cross-scale aggregation can help the network combine both fine details and broader context, which may improve boundary accuracy compared to using only same-level skips. The decoder-to-decoder skip connections (Dec-only configuration) also provide performance gains by facilitating progressive feature refinement across decoder levels. These lateral connections enable the propagation of refined features from deeper decoder layers to shallower ones, allowing for iterative improvement of segmentation predictions. The absence of these connections forces each decoder layer to work in isolation, preventing the beneficial exchange of refined semantic information and limiting the model’s ability to produce coherent, multi-scale predictions.

The superior performance of the complete ES-UNet architecture (“Both” configuration) validates our design philosophy that combining both skip connection types creates a synergistic effect. The encoder-to-decoder connections provide rich multi-scale input features, while the decoder-to-decoder connections enable progressive refinement of these features, resulting in more accurate and consistent segmentation outcomes.

#### Multi-component ablation study

Table 5 summarizes all the results shown in Sects. Simulation results for the ES-UNet architecture, Effect of the region specific scaling technique, and Effect of the DWD loss function. It also shows combined results when all technologies are used together. These ablation study results clearly demonstrate the incremental benefits of each proposed component across different architectures. For the baseline 3D UNet, adding RSS augmentation improved DSC by 1.22%, while incorporating DWD loss provided a 1.06% gain. When both components were combined, the improvement reached 2.17%, indicating a synergistic effect rather than merely additive benefits.

**Table 5:** Comparison of DSC, IoU and VOE with different configurations

Model	RSS	DWD Loss	DSC (%$\uparrow$)	IoU (%$\uparrow$)	VOE (%$\downarrow$)	$\Delta$DSC (%$\uparrow$)	$\Delta$IoU (%$\uparrow$)
3D UNet Ҫiçek et al. [23]			73.81	62.40	37.60	–	–
	$\checkmark$		75.03	63.57	36.43	1.22	1.17
		$\checkmark$	74.87	63.42	36.58	1.06	1.02
	$\checkmark$	$\checkmark$	**75.98**	**64.59**	**35.41**	**2.17**	**2.19**
3D UNet 3+ Huang et al. [17]			74.74	63.23	36.77	–	–
	$\checkmark$		75.17	63.94	36.06	0.43	0.71
		$\checkmark$	75.47	64.02	35.98	0.77	0.79
	$\checkmark$	$\checkmark$	**76.01**	**64.77**	**35.23**	**1.31**	**1.54**
ES-UNet			75.38	63.61	36.39	–	–
	$\checkmark$		75.74	64.59	35.41	0.36	0.98
		$\checkmark$	76.02	64.73	35.27	0.64	1.12
	$\checkmark$	$\checkmark$	**76.87**	**65.49**	**34.51**	**1.49**	**1.88**

Similarly, for 3D UNet 3+, RSS and DWD loss contributed improvements of 0.47% and 0.77% respectively, with their combination yielding a 1.31% enhancement. The proposed ES-UNet architecture itself outperformed the baseline models, achieving a DSC of 75.38% even without additional components. Adding RSS to ES-UNet provided a modest 0.36% improvement, while DWD loss contributed 0.64%. Most notably, the full ES-UNet model with both RSS and DWD loss achieved the highest overall performance with a DSC of 76.87%.

These results validate that each component makes a meaningful contribution to the overall performance gain. The observation that improvements are consistent across different architectures indicates the generalizability of both RSS augmentation and DWD loss. Furthermore, the varying magnitudes of improvement suggest that the baseline architectural differences influence how much benefit is derived from each component, with simpler architectures like 3D UNet gaining more from these enhancements compared to already-optimized structures like ES-UNet.

### Sensitivity analysis

#### Sensitivity analysis of the hyperparameter $\alpha$

Additionally, we conducted a sensitivity analysis for the hyperparameter $\alpha$ in Eq. 6, which controls the relative weight between focal loss and DWD loss components. As shown in Table 6, we evaluated three different $\alpha$ values (0.5, 1, and 2) using the full ES-UNet model with both RSS and DWD loss. The results demonstrate that $\alpha = 1$ achieves the optimal balance, yielding the highest DSC of 76.87%. While $\alpha = 0.5$ and $\alpha = 2$ show slightly lower performance (75.36% and 75.05% respectively), the differences are relatively modest, indicating that the proposed framework is reasonably robust to this hyperparameter choice.

**Table 6:** Ablation study of $\alpha$ in loss function

Alpha	DSC	IoU	VOE
	(%$\uparrow$)	(%$\uparrow$)	(%$\downarrow$)
$\alpha = 0.5$	75.36	64.26	35.74
$\alpha = 1$	**76.87**	**65.49**	**34.51**
$\alpha = 2$	75.05	63.70	36.30

#### Sensitivity analysis of the RSS scaling range

To further investigate the robustness of our RSS strategy, we performed a sensitivity analysis to examine how different scaling ranges influence segmentation performance. Three ranges were evaluated:

- A narrower range $\displaystyle\left[4/5,\,5/4\right]$ (smaller deformation)
- The base range $\displaystyle\left[2/3,\,3/2\right]$ (moderate deformation)
- A wider range $\displaystyle\left[1/2,\,2\right]$ (larger deformation)

The results, summarized in Table 7, show that the baseline range consistently achieved the highest Dice score (DSC 76.87%), outperforming the narrower (75.81%) and wider (74.32%) settings. When analyzing these results more deeply, we observed that each scaling range presents distinct trade-offs: The narrower range [$4/5$, $5/4$] introduces minimal distortion to the anatomical structures, maintaining high fidelity to the original images. However, this conservative approach provides insufficient variability in the training data, limiting the model’s ability to generalize to more diverse anatomical presentations. This explains the modest performance degradation (approximately 1% DSC reduction) compared to our original range. Conversely, the wider range [$1/2$, $2$] creates excessive deformation that, while increasing data diversity, tends to produce anatomically implausible transformations. These aggressive distortions can introduce artifacts and unrealistic spatial relationships between anatomical structures, particularly when applied along multiple axes simultaneously. This explains the more significant performance drop (approximately 2.5% DSC reduction) with this configuration. The original range [$2/3$, $3/2$] represents an optimal balance, introducing sufficient variability to enhance generalization while preserving anatomical plausibility, which confirms that our initial selection of scaling parameters was appropriate. These findings reinforce the effectiveness of our chosen scaling strategy and provide quantitative evidence that supports its use in anatomically diverse segmentation scenarios.

**Table 7:** Effect of RSS scaling ranges on DSC, IoU and VOE for ES-UNet

Range	Scaling Interval	DSC (%$\uparrow$)	IoU (%$\uparrow$)	VOE (%$\downarrow$)
Smaller Range	$[4/5,\,5/4]$	75.81	64.56	35.44
Base Range	$[2/3,\,3/2]$	**76.87**	**65.49**	**34.51**
Larger Range	$[1/2,\,2]$	74.39	62.68	37.32

### Comparison with state-of-the-art 3D segmentation models

To further assess the effectiveness of the proposed ES-UNet, we compared its performance with two state-of-the-art 3D segmentation architectures: nnUNet Isensee et al. [20] and Swin UNETR Hatamizadeh et al. [19]. These models represent distinct architectural paradigms in medical image segmentation: nnUNet is a self-configuring framework that automatically adapts its architecture to the dataset, while Swin UNETR leverages transformer-based attention mechanisms. Both have demonstrated strong performance across various medical imaging tasks and serve as important benchmarks in recent literature.

#### Performance comparison

Table 8 presents the comprehensive evaluation metrics for all models on the HECKTOR dataset. ES-UNet achieved the highest DSC of 76.87%, surpassing both Swin UNETR (76.02%) and nnUNet (76.06%), as well as the traditional UNet variants.

**Table 8:** Comparison with state-of-the-art models, nnUnet, swin UNETR and the proposed ES-UNet in terms of DSC, IoU and VOE

Model	DSC (%$\uparrow$)	IoU (%$\uparrow$)	VOE (%$\downarrow$)
nnUNet (3d) Isensee et al. [20]	76.06	65.46	34.54
Swin UNETR Hatamizadeh et al. [19]	76.17	65.17	34.83
ES-UNet	**76.87**	**65.49**	**34.51**

The superior performance of ES-UNet can be attributed to several key factors: Unlike nnUNet, which uses conventional skip connections, and Swin UNETR, which relies on transformer-based global attention, ES-UNet leverages enhanced full-scale skip connections inspired by UNet 3+. This design enables each decoder layer to aggregate features from all encoder levels simultaneously, preserving both fine-grained details and high-level semantic information. This comprehensive feature fusion is particularly effective for segmenting head and neck tumors, which often exhibit complex boundaries and heterogeneous texture patterns.

The proposed Dynamically Weighted Dice (DWD) loss adaptively balances precision and recall throughout training, automatically adjusting its focus based on the model’s current performance. This contrasts with nnUNet’s fixed loss combinations and Swin UNETR’s standard loss functions. The dynamic adjustment capability of DWD loss proves especially beneficial for handling the inherent class imbalance in tumor segmentation and capturing irregular tumor boundaries more precisely.

Our RSS augmentation technique specifically targets the region of interest, providing more meaningful variations compared to standard augmentation strategies used in other methods. This targeted approach enhances the model’s robustness to anatomical variations without compromising the integrity of surrounding structures.

#### Computational efficiency analysis

Table 9 presents a comprehensive analysis of computational complexity across all evaluated models. Building on 3D UNet 3+, ES-UNet enhances its full-scale skip connections by incorporating lightweight channel attention on each encoder-to-decoder path and employs learnable transposed convolution layers for upsampling between adjacent decoder stages. This configuration offers a balanced trade-off between detailed feature preservation and computational demands: it enhances feature refinement and boundary recovery at the cost of a modest increase in parameters, while FLOPs, inference speed, and GPU memory usage remain competitive or slightly improved. In contrast, when compared to more recent models such as nnUNet and Swin UNETR, the opposite trend is observed. While ES-UNet shows a smaller parameter count, owing to its relatively compact architecture without transformers or auto-configured modules, it exhibits higher computational demands in terms of FLOPs and memory usage due to our full-scale feature integration strategy.

**Table 9:** Comparison of computational complexity on HECKTOR dataset

Model	Params(M)	FLOPs(G)	Inference speed(ms/sample)^†^	Peak memory(GB)
3D UNet Ҫiçek et al. [23]	6.42	794.57	60.41	5.30
3D UNet 3+ Huang et al. [17]	6.14	3286.40	184.91	16.77
nnUNet (3d) Isensee et al. [20]	31.20	480.83	33.61	3.71
Swin UNETR Hatamizadeh et al. [19]	70.15	772.92	122.82	12.07
ES-UNet	9.01	3020.38	178.52	16.13

†Measured on a single NVIDIA RTX 4090 (24 GB) GPU

As shown in Table 9, the proposed ES-UNet architecture presents mixed computational efficiency results, showing advantages in some aspects while requiring more resources in others depending on which metric is prioritized. However, in clinical applications where diagnostic accuracy is paramount and offline processing is acceptable, segmentation performance remains the primary consideration. As shown in Tables 8 and 10, ES-UNet consistently achieves superior segmentation performance across diverse evaluation scenarios. Our method outperforms traditional UNet variants (3D UNet, 3D UNet 3+) with substantial DSC improvements, demonstrating the effectiveness of our architectural enhancements. More importantly, ES-UNet also surpasses recent state-of-the-art methods, achieving 76.87% DSC compared to 76.06% for nnUNet and 76.17% for Swin UNETR on the HECKTOR dataset. Taken together, ES-UNet offers a well-balanced and effective solution for high-precision 3D medical image segmentation tasks, particularly in real-world settings where diagnostic quality is more important than absolute computational minimization.

**Table 10:** Segmentation performance on HECKTOR and MSD tasks

Model	HECKTOR		MSD Heart		MSD Spleen	
	DSC (%$\uparrow$)	VOE (%$\downarrow$)	DSC (%$\uparrow$)	VOE (%$\downarrow$)	DSC (%$\uparrow$)	VOE (%$\uparrow$)
nnUNet (3d) Isensee et al. [20]	76.06	34.54	89.83	18.31	89.66	16.70
Swin UNETR Hatamizadeh et al. [19]	76.17	37.83	89.52	18.86	90.55	17.03
ES-UNet	**76.87**	**34.51**	**91.35**	**15.82**	**91.55**	**15.45**

### Cross-dataset evaluation

To evaluate the generalizability of the proposed ES-UNet beyond the HECKTOR dataset, we additionally tested the model on two datasets from the Medical Segmentation Decathlon (MSD): the Heart and Spleen datasets. The MSD Heart dataset includes 30 cine-MRI scans with annotations of the left atrium, a thin-walled structure with irregular boundaries and substantial anatomical variability across subjects. A total of 30 volumes in the Heart dataset consists of 20 labeled volumes for training and 10 unlabeled volumes for official testing. For our experiments, the 20 labeled training volumes were randomly split using a fixed random seed into 16 volumes for optimization and 4 volumes for evaluation, following the same 80:20 split strategy used for the HECKTOR dataset. All volumes were resized to $128 \times 128 \times 128$ voxels for standardized network input without cropping, ensuring that the complete anatomical context was preserved while maintaining computational efficiency.

The MSD Spleen dataset comprises 61 portal-venous-phase CT images, offering a different segmentation challenge with more clearly defined organ boundaries but variable organ appearances due to individual anatomical differences. A total of 61 volumes in the Spleen dataset consists of 41 labeled volumes for training and 20 unlabeled volumes for official testing. Following the same methodology as the Heart dataset, the 41 labeled training volumes were randomly split using the same fixed random seed into 33 volumes for optimization and 8 volumes for evaluation. All volumes were preprocessed using the identical pipeline, being resized to $128 \times 128 \times 128$ voxels without cropping to maintain consistency across all experiments. By applying the same preprocessing and training protocol across these different datasets, we aimed to fairly assess the generalizability of ES-UNet across varying anatomical structures and dataset characteristics.

Table 10 presents the comparative performance of ES-UNet against state-of-the-art models (nnUNet and Swin UNETR) across all three datasets. All models were trained and evaluated on the same fixed train/validation split, with no post-processing or ensembling, to ensure a fair comparison between architectures. As summarized in the table, ES-UNet consistently outperformed both nnUNet and Swin UNETR across all datasets, achieving the highest DSC and the lowest VOE in every case. Notably, the performance gains were more pronounced on the MSD Heart and Spleen datasets. One contributing factor may be the relatively small training size of these datasets compared to HECKTOR, which makes our RSS data augmentation strategy more impactful due to the greater need for variability. These results suggest that ES-UNet not only performs well on anatomically complex tumor segmentation (e.g., HECKTOR) but also generalizes effectively to other organ-level tasks with varying structural and data characteristics.

### 3D visualization of segmentation results

**Fig. 9:**
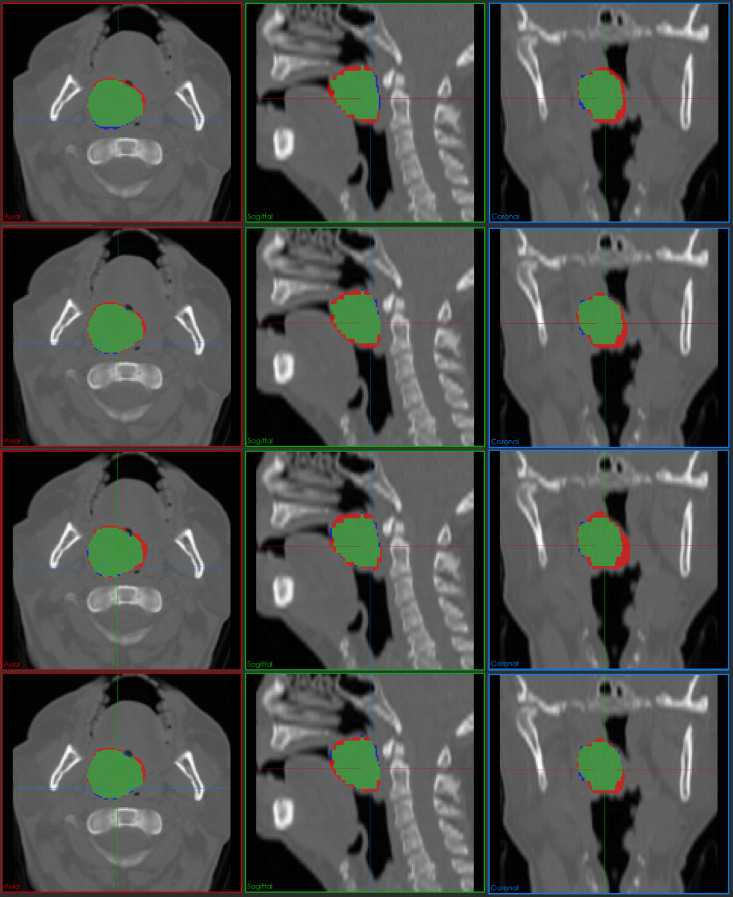
Comparative segmentation results on the HECKTOR dataset. Four rows show results from 3D UNet, nnUnet, Swin UNETR, and ES-UNet, respectively. Color coding: green (true positive), red (false positive), blue (false negative). Three columns represent axial, sagittal, and coronal views, respectively

**Fig. 10:**
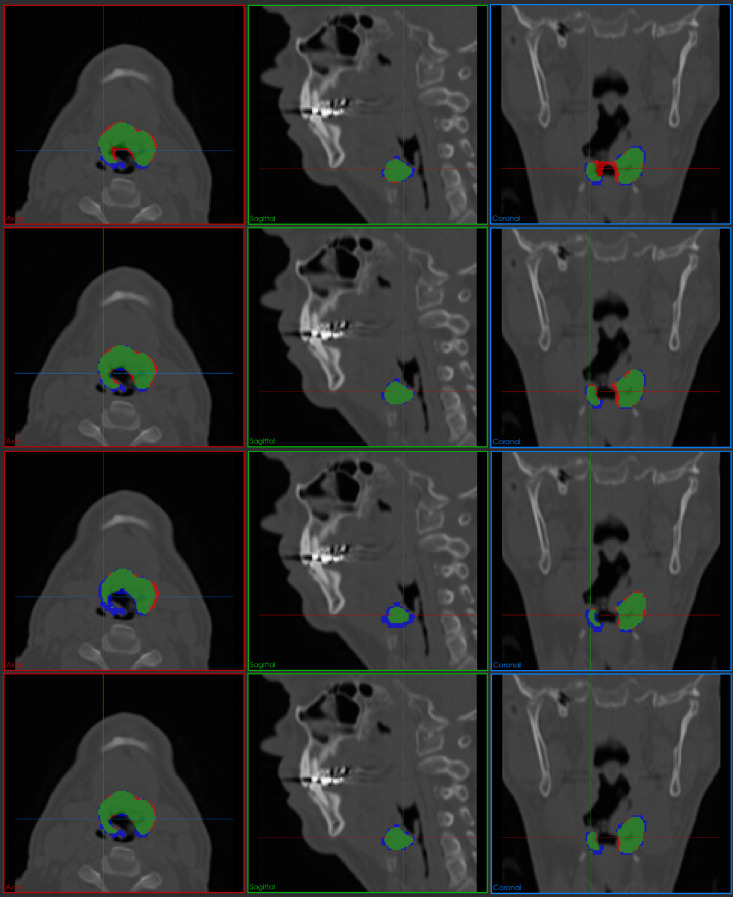
Comparative segmentation results on a challenging HECKTOR case with complex tumor morphology. Four rows show results from 3D UNet, nnUnet, Swin UNETR, and ES-UNet, respectively. Color coding: green (true positive), red (false positive), blue (false negative). Three columns represent axial, sagittal, and coronal views, respectively

To provide comprehensive visual validation of our quantitative results, we present detailed qualitative comparisons between ES-UNet and other methods including 3D UNet, nnUNet, and Swin UNETR. Figures 9 and 10 demonstrate representative segmentation results from the HECKTOR dataset across three orthogonal planes, axial, sagittal, and coronal views, following standard practices in 3D medical image analysis. The visual comparisons employ a consistent color-coding scheme where green indicates true positives (correctly segmented regions), red represents false positives (incorrectly segmented regions), and blue denotes false negatives (missed target regions).

Figure 9 presents segmentation results for a representative case from the HECKTOR dataset, featuring a head and neck tumor with relatively simple, convex morphology. For such geometrically straightforward cases, all four methods demonstrate reasonably good segmentation performance, successfully capturing the overall tumor structure. However, upon closer examination, subtle but meaningful differences emerge in segmentation precision. While 3D UNet, nnUNet, and Swin UNETR all achieve acceptable results, they exhibit varying degrees of false positive and false negative regions, particularly visible as red and blue artifacts in the boundary areas. ES-UNet demonstrates the most refined performance, with notably smaller false positive and false negative regions across all three anatomical planes. This improvement can be attributed to the synergistic effect of the full-scale attention-enhanced skip connections and the dynamically balanced DWD loss, which jointly enhance both feature propagation and error sensitivity during training.

Figure 10 presents a more challenging case from the HECKTOR dataset, where the target tumor exhibits significantly more complex, irregular morphology with intricate boundary patterns. Such complexity often amplifies the performance gap between segmentation methods, as they require sophisticated feature learning capabilities to accurately delineate irregular boundaries and handle structural heterogeneity. The comparative analysis in Fig. 10 clearly demonstrates these expected performance variations. The 3D UNet tends to over-segment into adjacent tissues, particularly in the coronal view, leading to a significant number of FP voxels. Although nnUNet shows improved performance compared to 3D UNet, it still produces a considerable amount of over-segmentation in the coronal view, yielding a non-negligible number of false positives. Meanwhile, the Swin UNETR shows substantial FN regions across all axial, sagittal, and coronal views, indicating its failure to capture the fine extensions of the lesion. On the other hand, ES-UNet demonstrates robust shape conformity with substantially reduced FP and FN regions, reflecting its ability to better capture spatial context and preserve anatomical plausibility.

These visual comparisons reaffirm the advantages of the proposed ES-UNet in producing compact, accurate, and anatomically consistent segmentations, particularly in geometrically complex cases. The qualitative trends observed here are consistent with the quantitative results in Tables 5 and 8 discussed earlier, providing further validation for the effectiveness of the proposed architectural and algorithmic improvements.

To provide comprehensive demonstration of our 3D segmentation capabilities, we present volumetric 3D visualizations that demonstrate the true three-dimensional nature of our segmentation results. While 2D slice-based comparisons are helpful for assessing segmentation quality in specific anatomical planes, 3D visualizations provide a more comprehensive understanding of three-dimensional morphology and structural fidelity across the entire volume. Especially in medical imaging applications where lesions exhibit complex 3D morphology, visual inspection of full volumes plays a crucial role in evaluating clinical usability.

**Fig. 11:**
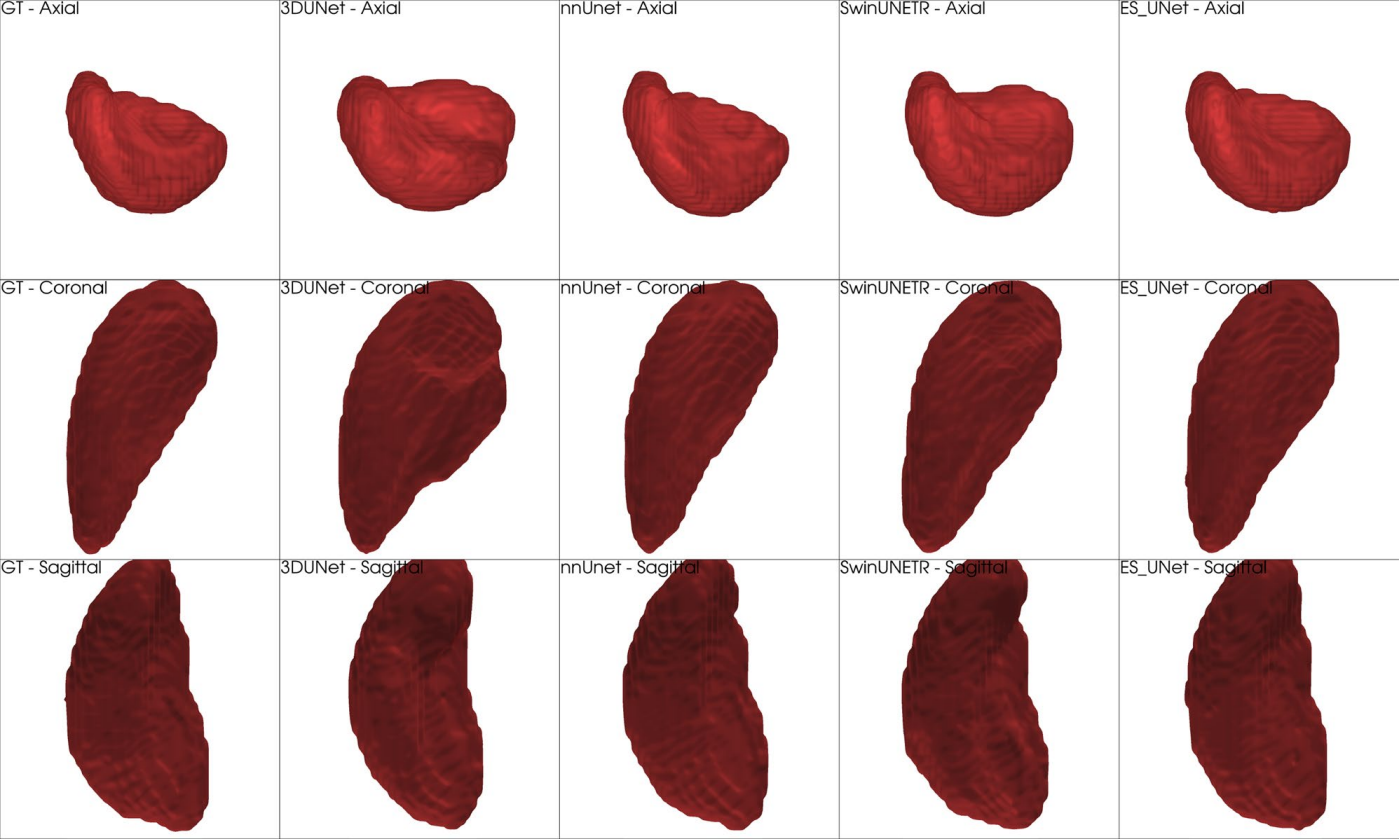
3D volumetric comparison of spleen segmentation results from the MSD dataset. From left to right: ground Truth, 3D UNet, nnUnet, Swin UNETR, and ES-UNet results. Three rows represent axial, coronal, and sagittal viewing perspectives, respectively

Figure 11 presents 3D volumetric renderings of spleen segmentation results from the MSD Spleen dataset, comparing our proposed ES-UNet with three other segmentation methods across three anatomical viewing angles (axial, coronal, and sagittal perspectives). The overall shape of the spleen in this case is relatively simple and well-defined, leading to comparable performance across all models. However, despite the generally accurate segmentations, 3D UNet shows clear signs of over-segmentation in the coronal view, where the predicted region spreads into adjacent non-splenic areas. In contrast, nnUNet, Swin UNETR, and ES-UNet maintain a more compact and anatomically plausible shape. Although performance differences are less pronounced in this case, ES-UNet still demonstrates clean boundaries with minimal structural distortion.

**Fig. 12:**
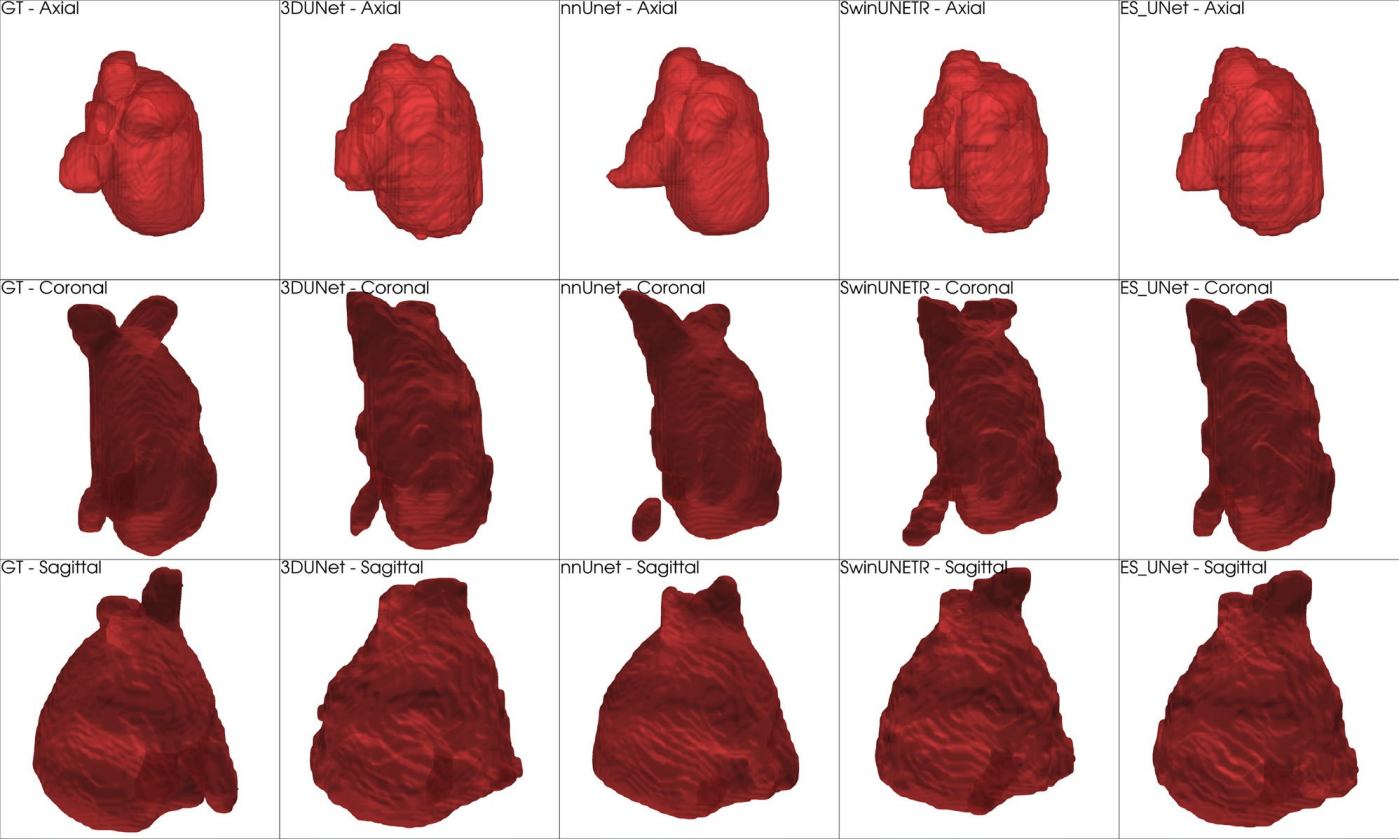
3D volumetric comparison of heart (left atrium) segmentation results from the MSD dataset. From left to right: ground Truth, 3D UNet, nnUnet, Swin UNETR, and ES-UNet results. Three rows represent axial, coronal, and sagittal viewing perspectives, respectively

Figure 12 shows 3D volumetric renderings of left atrium segmentation from the MSD Heart dataset, presenting a significantly more complex segmentation challenge. The left atrium, with its thin-walled, irregular structure, represents one of the most demanding anatomical structures for accurate 3D segmentation. Unlike the relatively smooth spleen morphology, cardiac structures typically exhibit complex geometries that require sophisticated feature learning capabilities.

The comparative analysis clearly demonstrates these increased segmentation challenges across all methods. The 3D UNet results show substantial segmentation errors, including missed regions and inaccurate boundary delineation. The nnUNet demonstrates improved performance but exhibits concerning artifacts, notably the presence of disconnected segmented regions visible in the lower left area of the coronal view, suggesting incomplete connectivity understanding. Additionally, Swin UNETR also exhibits over-segmentation artifacts, particularly visible in the lower left area of the coronal view where false positive regions are noticeably larger compared to other methods.

On the other hand, ES-UNet consistently maintains superior segmentation accuracy across all viewing angles, effectively capturing the complex three-dimensional morphology of the left atrium. Most notably, our method demonstrates excellent conformity to the ground truth structure, particularly in challenging regions such as the complex upper portions visible in coronal and sagittal views where anatomical topology becomes intricate. The enhanced accuracy in these demanding areas can be attributed to our full-scale feature integration approach, which preserves fine-grained structural information from multiple encoder levels simultaneously, and our Dynamically Weighted Dice (DWD) loss function, which adaptively balances precision and recall during training. The quantitative results presented in Table 10 strongly support these qualitative observations. These quantitative gains translate into clinically meaningful improvements in 3D reconstruction accuracy, demonstrating that ES-UNet’s architectural enhancements effectively leverage the full spatial context available in volumetric medical data for robust and accurate organ segmentation across diverse anatomical structures.

## Discussion

The results of this study demonstrate that the proposed ES-UNet architecture, combined with the Region Specific Scaling (RSS) data augmentation technique and the Dynamically Weighted Dice (DWD) loss function, improves the accuracy of 3D medical image segmentation compared to existing models such as 3D UNet and 3D UNet 3+, as well as state-of-the-art approaches like nnUNet and Swin UNETR. This performance improvement stems from key innovations in the architecture and learning strategies, addressing challenges such as data scarcity, class imbalance, and the need for precise localization in medical image segmentation.

Firstly, the ES-UNet architecture enhances skip connections to improve feature propagation and model robustness while managing computational demands. While UNet 3+ employs dense skip connections for multi-scale feature fusion, ES-UNet preserves all of those encoder-to-decoder paths and enhances each one with lightweight channel attention, while also introducing learnable transposed convolution layers between adjacent decoder stages for improved upsampling. At the same time, it ensures that each decoder block receives balanced multi-scale feature information. Additionally, the introduction of a channel attention mechanism allows the model to focus on the most important features in volumetric data without increasing the complexity of intermediate structures, thereby improving segmentation accuracy. Full-scale deep supervision also contributes to performance enhancement by guiding the model to learn robust representations at various levels.

Secondly, the RSS data augmentation technique addresses the issue of insufficient training data, which is common in medical imaging. Traditional augmentation methods like flipping and rotation often produce samples that are too similar to the originals, limiting their effectiveness in preventing overfitting. In contrast, RSS scales the region of interest (e.g., tumors) along the height, width, and depth axes within a specific ratio while preserving anatomical integrity, thereby generating diverse training samples. This improves the model’s generalization ability, as confirmed by the results in Table 2, where RSS enhanced DSC across all tested models. While our experiments demonstrate that the fixed scaling range of [$2/3$, $3/2$] provides an optimal balance between introducing meaningful variations and preserving anatomical plausibility for the datasets we tested, further research could explore adaptive scaling techniques. Such approaches might automatically determine optimal scaling parameters based on dataset-specific anatomical characteristics, potentially through learning-based methods that analyze the spatial statistics of target structures. This adaptive approach could further enhance the effectiveness of RSS across diverse anatomical structures with varying shapes, sizes, and spatial relationships.

Thirdly, the DWD loss function enhances the accuracy of medical image segmentation by dynamically balancing precision and recall. The DWD loss adaptively adjusts its weighting factors based on the model’s current performance, allowing it to focus on improving either precision or recall depending on which aspect requires more attention during different stages of training. This adaptive approach is particularly valuable for handling imbalanced classes and complex boundary definitions common in medical image segmentation tasks.

Our cross-dataset evaluation on MSD Heart and MSD Spleen datasets further validates the generalizability of our approach. The consistent performance improvements across three distinct anatomical structures (head and neck tumors, heart, and spleen) with different imaging characteristics provides evidence that ES-UNet’s enhancements extend beyond any specific dataset or anatomical region. This cross-validation demonstrates the robustness of our method across diverse medical segmentation tasks.

However, this study has several limitations. First, RSS uses a manually set scaling ratio within a predefined range [$2/3$, $3/2$], which may not be optimal for all anatomical structures or lesions. Future research should explore adaptive scaling techniques that adjust based on the characteristics of the target region.

Second, there is room to explore multi-modal fusion strategies. While this study used PET and CT images as input channels, more sophisticated methods such as attention-based fusion or modality-specific encoders could further improve segmentation performance. A promising direction is to explicitly exploit the complementary nature of each modality during both feature extraction and integration. For instance, recent work by Li et al. [32] introduced a modality fusion module and cross-modality-assisted skip connections to integrate multiple MRI sequences (e.g., T1, T2, FLAIR) in brain tumor segmentation, achieving robust performance even when dominant modalities were unavailable. Their approach highlights the benefits of learning complementary representations across modalities in semi-supervised settings. Similarly, Jin et al. [33] proposed a multi-modality contrastive learning framework that combines hip X-ray images with clinical parameters, demonstrating improved performance in sarcopenia screening. These approaches suggest that incorporating advanced multimodal fusion techniques into our ES-UNet architecture could yield significant performance improvements, particularly for complex anatomical structures where different modalities provide complementary diagnostic information.

Third, semi-supervised learning is another promising direction, particularly in scenarios with limited labeled data. Recent research has proposed innovative ways to utilize unlabeled data through consistency learning and uncertainty modeling. For example, Li and Xie [34] proposed EBC-Net, a 3D semi-supervised framework that employs edge-biased consistency regularization and anatomical invariance modules to guide pancreas segmentation with minimal supervision. Their approach improves boundary accuracy in complex organ structures and demonstrates the effectiveness of edge-aware perturbation strategies. Other recent works Jin et al. [35, 36] have explored hierarchical consistency enforcement and uncertainty-aware pseudo-mask refinement to boost segmentation performance. Integrating such semi-supervised techniques into 3D segmentation frameworks like ours may enable more robust learning in low-resource settings and expand applicability to real-world clinical scenarios.

In addition, the proposed DWD loss may not always perform optimally in certain scenarios. For instance, in datasets where class distributions are relatively balanced, the benefit of dynamic weighting becomes marginal, and simpler loss functions such as Dice or cross-entropy may suffice. Moreover, during the early phases of training, when predictions are highly unstable, DWD’s reliance on real-time precision and recall estimates can introduce noisy gradients, potentially hindering convergence. Another limitation arises in multi-class segmentation tasks, where the dynamic weighting mechanism requires additional design considerations for per-class adaptation, making its implementation less straightforward than that of generalized Dice. These limitations highlight the need for further investigation, and we plan to explore more robust extensions of DWD for multi-class scenarios and early-stage stabilization strategies in future work.

## Conclusions

This study proposed ES-UNet, a novel 3D segmentation architecture that integrates architectural refinements with advanced training strategies to address key challenges in medical image segmentation. The model improves upon prior UNet-based methods by introducing lightweight channel attention on every encoder-to-decoder path, while keeping UNet3+’s full-scale skip connections intact. Additionally, learnable transposed convolution layers are employed between adjacent decoder stages to enhance boundary reconstruction during upsampling. These design choices aim to enhance feature learning and segmentation performance while managing computational demands. Through extensive evaluations on the HECKTOR dataset and selected tasks from the Medical Segmentation Decathlon, ES-UNet demonstrated strong segmentation accuracy and generalization ability across different anatomical regions.

Compared to prior UNet variants, ES-UNet improves both accuracy and computational efficiency in some areas, while requiring slightly more resources in others. Against more recent models like nnUNet and Swin UNETR, it achieves superior segmentation accuracy, though not always with lower computational cost. Importantly, in offline clinical settings where diagnostic accuracy is the primary concern, we believe ES-UNet offers a favorable trade-off between performance and resource requirements. Its ability to operate directly on full 3D volumes enables it to capture spatial context more effectively than slice-based methods, contributing to its robust performance.

Overall, ES-UNet demonstrates that targeted architectural refinements, when combined with targeted augmentation (RSS) and adaptive loss functions (DWD), can lead to practical and high-performing 3D segmentation models. Future research will focus on developing adaptive scaling techniques for the RSS method, exploring advanced fusion strategies to better integrate multi-modal information, and extending the framework to semi-supervised settings where labeled data is limited.

## Data Availability

The datasets used in this study include the MICCAI Head and Neck Tumor Segmentation (HECKTOR) dataset, which is publicly available at https://hecktor.grand-challenge.org, and two datasets from the Medical Segmentation Decathlon (MSD), the Spleen and Heart tasks, available at http://medicaldecathlon.com.

## Abbreviations

AI: Artificial Intelligence
CEL: Cross Entropy Loss
CNN: Convolutional Neural Network
DWD: Dynamically Weighted Dice
CT: Computed Tomography
DSC: Dice Similarity Coefficient
HECKTOR: Head and Neck Tumor Segmentation
IoU: Intersection over Union
MRI: Magnetic Resonance Imaging
PET: Positron Emission Tomography
RSS: Region Specific Scaling
SENet: Squeeze and Excitation Network
VOE: Volume Overlap Error
