# Correction: ES-UNet: efficient 3D medical image segmentation with enhanced skip connections in 3D UNet

**DOI:** 10.1186/s12880-025-01891-y

**Published:** 2025-09-02

**Authors:** Minyoung Park, Seungtaek Oh, Junyoung Park, Taikyeong Jeong, Sungwook Yu

**Affiliations:** 1 School of Electrical and Electronics Engineering, Chung-Ang University, 84 Heukseok-Ro, Dongjak-Gu, Seoul, 06974 Republic of Korea; 2 School of Artificial Intelligence Convergence, Hallym University, 1 Hallymdaehak-Gil, Chuncheon, 24252 Republic of Korea

**Correction: Park et al. BMC Medical Imaging (2025) 25:327**

10.1186/s12880-025-01857-0

Following the publication of the original article, the authors reported a symbol ($) were erroneously introduced during the proofreading. The correct table is as follows:

**Table:** 

Model	Params(M)	FLOPs(G)	Inference speed(ms/sample)†	Peak memory(GB)
3D UNet Ҫiçek et al. [23]	6.42	794.57	60.41	5.3
3D UNet 3 + Huang et al. [17]	6.14	3286.4	184.91	16.77
nnUNet (3d) Isensee et al. [20]	31.2	480.83	33.61	3.71
Swin UNETR Hatamizadeh et al. [19]	70.15	772.92	122.82	12.07
ES-UNet	9.01	3020.38	178.52	16.13

†Measured on a single NVIDIA RTX 4090 (24 GB) GPU

Moreover, the layout of Table 2 is revised to improve readability.

The original article has been updated accordingly.

